# Supplementary figures and images for: Glucose-6-phosphate dehydrogenase activity in individuals with and without malaria: Analysis of clinical trial, cross-sectional and case–control data from Bangladesh
Source: PLoS Med. 2021 Apr 23;18(4):e1003576. doi: 10.1371/journal.pmed.1003576 (PMC8064587; doi:10.1371/journal.pmed.1003576)

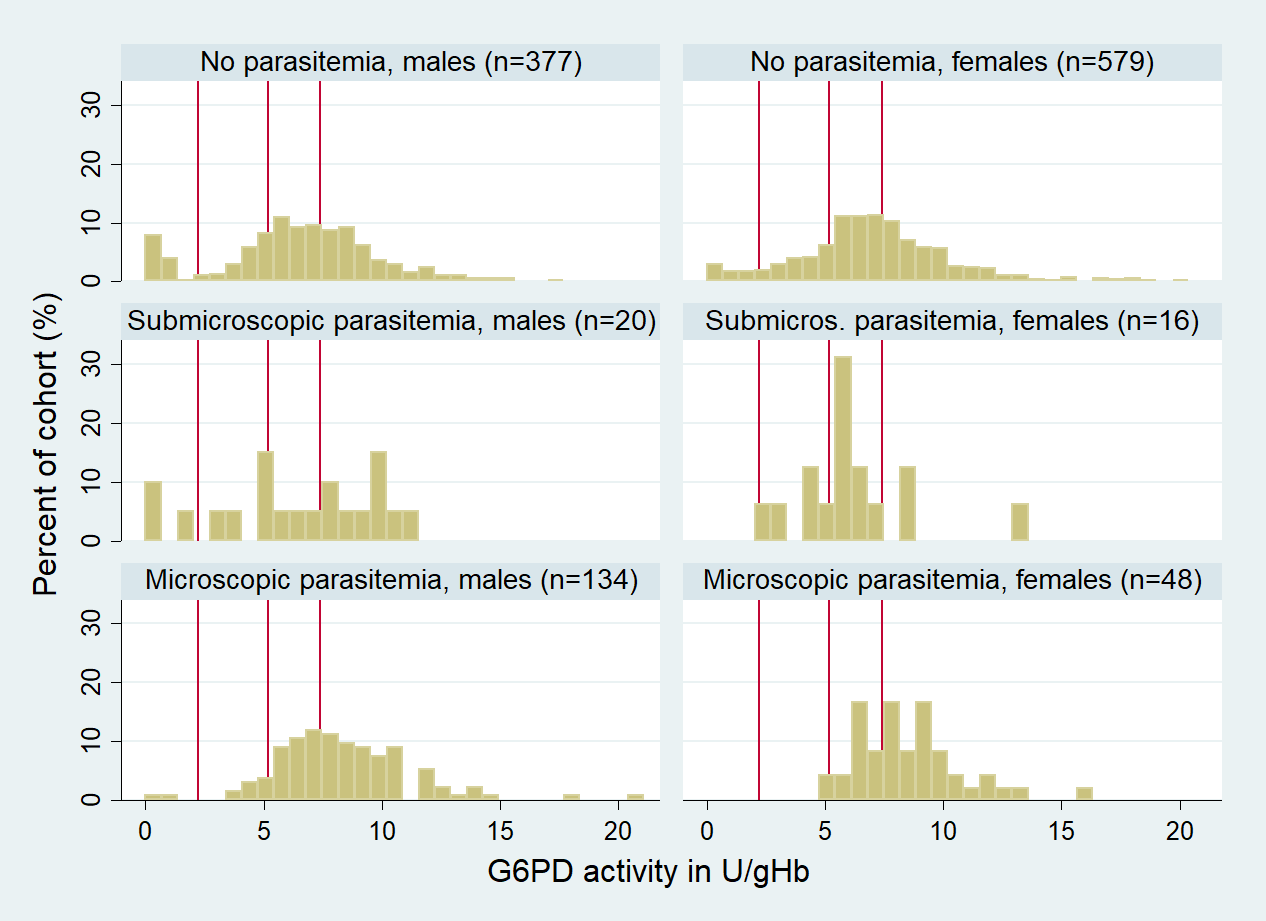

Supplement: S1 Fig — Red vertical lines indicate 30%, 70%, and 100% G6PD activity of the adjusted male median. 100% G6PD activity = 7.4 U/g Hb. (TIF) [file pmed.1003576.s001.tif]
