# Supplementary material for: Glucose-6-phosphate dehydrogenase activity in individuals with and without malaria: Analysis of clinical trial, cross-sectional and case–control data from Bangladesh
Source: PLoS Med. 2021 Apr 23;18(4):e1003576. doi: 10.1371/journal.pmed.1003576 (PMC8064587; doi:10.1371/journal.pmed.1003576)
Supplement: S1 Table — (DOCX) [file pmed.1003576.s002.docx]

| **Study** | **Protocol name** | **HREC number (Australia)** | **ERC number (Bangladesh)** |
| --- | --- | --- | --- |
| Efficacy trial | A study to assess primaquine treatment guidelines on malaria in South-East Bangladesh | 2014-2228 | PR-14053 |
| Cross-sectional survey | Prevalence of G6PD deficiency and *G6PD* variants among the indigenous population of the Bandarban District, Chittagong Hill Tracts, Bangladesh (GPAT) | 2015-2336 | PR-15021 |
| Case-control study | Community and facility assessment to determine populations at risk of malaria and primaquine induced haemolysis (ACROSS) | 2017-3010 | PR-18001 |
