# Supplementary material for: Glucose-6-phosphate dehydrogenase activity in individuals with and without malaria: Analysis of clinical trial, cross-sectional and case–control data from Bangladesh
Source: PLoS Med. 2021 Apr 23;18(4):e1003576. doi: 10.1371/journal.pmed.1003576 (PMC8064587; doi:10.1371/journal.pmed.1003576)
Supplement: S2 Table — (DOCX) [file pmed.1003576.s003.docx]

| **Variant** | **n (%)** | **Median G6PD activity (IQR, range)** |
| --- | --- | --- |
| Mahidol hemi/homozygous | 58 (16.7) | 0.51  IQR: 0.14 – 0.78  Range: 0.00 – 13.06 |
| Mahidol heterozygous | 43 (12.4) | 3.78  IQR: 2.06 – 5.07  Range: 0.00 – 14.52 |
| Orissa hemi/homozygous | 7 (2.0) | 2.69  IQR: 0.70 – 3.17  Range: 0.27 – 4.19 |
| Orissa heterozygous | 1 (0.3) | 6.22 |
| Kaylan – Kerala hemizygous | 1 (0.3) | 2.07 |
| No variant | 238 (68.4) | 4.76  IQR: 3.68 – 6.88)  Range: 0.00 – 16.53 |
| Total | 348 (100.0) | 4.19  IQR: 2.04 – 6.17)  Range: 0.00 – 16.53 |
