# Supplementary material for: Glucose-6-phosphate dehydrogenase activity in individuals with and without malaria: Analysis of clinical trial, cross-sectional and case–control data from Bangladesh
Source: PLoS Med. 2021 Apr 23;18(4):e1003576. doi: 10.1371/journal.pmed.1003576 (PMC8064587; doi:10.1371/journal.pmed.1003576)
Supplement: S3 Table — (DOCX) [file pmed.1003576.s004.docx]

| **G6PD variant** | **Cases** | | **Control** | |  |
| --- | --- | --- | --- | --- | --- |
|  | **n (% per column)** | **Median G6PD activity in U/gHb (IQR, min - max)** | **n (% per column)** | **Median G6PD activity in U/gHb (IQR, min-max)** | ***p*** |
| **Mahidol homozygous** | 11 (68.8) | 0.96 (0.46 – 1.68, 0.25 – 1.95) | 17 (73.9) | 0.88 (0.67 – 1.04, 0.54 – 1.72) | 0.725 |
| **Mahidol heterozygous** | 5 (31.3) | 4.20 (3.95 – 5.35, 3.85 – 6.03) | 2 (8.7) | 6.61 (6.51 – 6.70, 6.51 – 6.70) | 0.071 |
| **Mediterranean heterozygous** | 0 (0.0) | n=0 | 1 (4.4) | 2.34 (n=1) | 0.398 |
| **Orissa heterozygous** | 0 (0.0) | n=0 | 3 (13.0) | 1.54 (1.33 – 7.44, 1.33 – 7.44) | 0.133 |
| **Total** | 16 (100.0) | - | 23 (100.0) | - | - |
