# Supplementary material for: Glucose-6-phosphate dehydrogenase activity in individuals with and without malaria: Analysis of clinical trial, cross-sectional and case–control data from Bangladesh
Source: PLoS Med. 2021 Apr 23;18(4):e1003576. doi: 10.1371/journal.pmed.1003576 (PMC8064587; doi:10.1371/journal.pmed.1003576)
Supplement: S1 File — (DOCX) [file pmed.1003576.s005.docx]

STROBE Statement—checklist of items that should be included in reports of observational studies

|  | Item No | Recommendation | Location |
| --- | --- | --- | --- |
| **Title and abstract** | 1 | Variability of Glucose-6-Phosphate Dehydrogenase activity in individuals with and without malaria in the Chittagong Hill Tracts of Bangladesh: combining results of a treatment efficacy trial, cross-sectional survey, and a case-control study | Start of article |
|  |  | Background: Glucose-6-phosphate dehydrogenase (G6PD) activity is dependent upon G6PD genotype and age of the red blood cell (RBC) population, with younger RBCs having higher activity. Peripheral parasitemia with Plasmodium spp. induces hemolysis, replacing older RBCs with younger cells with higher G6PD activity. This study aimed to assess whether G6PD activity varies between individuals with and without malaria or a history of malaria.  Methods and Findings: Individuals, with and without malaria, living in the Chittagong Hill Tracts of Bangladesh were enrolled into three complementary studies: i) a clinical efficacy trial of patients with uncomplicated malaria, ii) a cross-sectional survey and iii) a matched case-control study of aparasitemic individuals with and without a history of malaria. G6PD activity was compared between individuals with and without malaria diagnosed by microscopy, rapid diagnostic test (RDT) or polymerase chain reaction (PCR) and also in aparasitaemic participants with and without a history of malaria. In the cross-sectional survey and clinical trial, 15.5% (182/1174) of participants had peripheral parasitaemia detected by microscopy or RDT, 3.1% (36/1174) were positive by PCR only and 81.4% (956/1174) were aparasitaemic. Individuals without malaria had significantly lower G6PD activity (median: 6.9 U/gHb (interquartile range (IQR):5.2 to 8.6)), compared to those with peripheral parasitaemia detected by microscopy or RDT (7.9 U/gHb, IQR: 6.6 to 9.8, p<0.001), but similar to those with low-level parasitaemia detected by PCR alone (6.1 U/gHb, IQR: 4.8 to 8.6, p=0.312). In total 7.7% (14/182) patients with malaria had G6PD activity <70% compared to 25.0% (248/992) of participants with submicroscopic or no parasitemia (Odds Ratio OR=0.25 [95%CI 0.14-0.44]); p<0.0001).  In the case control study, the median G6PD activity was 10.3 U/gH (IQR: 8.8 to 12.2) in the 253 patients with a history of malaria and 10.2 U/gHb (IQR: 8.7 to 11.8) in the 253 individuals without a history of malaria (p=0.323). The proportion of individuals with less than 70% activity was 11.5% (29/253) in the cases and 15.4% (39/253) in the controls (OR=0.7 [95%CI 0.41 – 1.23]; p=0.192). Limitations of the study included the non-contemporaneous nature of clinical trials and cross-sectional surveys.  Conclusions: Patients with acute malaria had significantly higher G6PD activity than individuals without malaria and this could not be accounted for by a protective effect of G6PD deficiency. These findings suggest that G6PD deficient patients with malaria may have higher than expected G6PD enzyme activity and an attenuated risk of primaquine-induced haemolysis compared to the risk when not infected. | Start of article - Abstract |
| Introduction | | | |
| Background/rationale | 2 | G6PD activity is dependent on age of a patient’s RBC population. Malaria induces haemolysis in a patient that results in erythropoiesis. In consequence the age of the RBC population of the patient decreases and G6PD activity should increase. This effect has not been described in detail to date | Introduction: 3^rd^ paragraph |
| Objectives | 3 | The aim of this study was to determine whether G6PD activity varies in individuals with and without malaria and whether this was attributable to a reduced risk of malaria. | Introduction: 3^rd^ paragraph |
| Methods | | | |
| Study design | 4 | Individuals were enrolled into three studies at the same location in the Chittagong Hill Tracts (CHT), Bangladesh: a clinical trial of patients presenting with uncomplicated P. falciparum or P. vivax malaria (18), a cross-sectional survey (19), and a case-control study. First, we determined whether G6PD activity differed between patients with symptomatic malaria, recruited from the efficacy trial, and afebrile and malaria negative participants from the cross-sectional survey. We then compared G6PD activities between participants with and without a history of malaria, all of whom were afebrile and free of malaria at the time point of G6PD measurement. | Methods: 2^nd^ paragraph |
| Setting | 5 | The clinical trial was conducted between September 2014 and February 2015 in Alikadam in the district of Bandarban (CHT). The cross-sectional survey was conducted in the area around Alikadam, district of Bandarban, between August 2015 and January 2016. In 2009, a prospective demographic surveillance system (DSS) was established in the Bandarban district amongst a population of 20,558, the DSS is ongoing. | Methods: 3^rd^, 4^th^ and 5^th^ paragraph |
| Participants | 6 | *Clinical trial:* Patients with uncomplicated malaria diagnosed by microscopy were enrolled  *Cross-sectional survey:* Resident of the study area  *Case control study:* Cases were selected randomly from DSS participants who had had at least one episode of malaria confirmed by microscopy during their monitoring within the DSS. Controls were selected from participants with no recorded episodes of malaria during their monitoring within the DSS. Cases and controls were selected in a ratio of 1:1, and matched according to the duration under surveillance, sex, age, and village of residence. | Methods: 3^rd^, 4^th^ and 5^th^ paragraph |
| Variables | 7 | - G6PD activity in U/gHb and U/dL - Malaria status by   - Microscopy / RDT   - PCR - History of malaria - Genotype | Methods: statistical analysis |
| Data sources/ measurement | 8* | - G6PD activity in U/gHb and U/dL: spectrophotometry - Malaria status by   - Microscopy / RDT   - PCR - History of malaria: through existing DSS database - Genotype: PCR | Methods: Statistical analysis |
| Bias | 9 | The results from Randox control samples generated from the clinical and survey participants were compared to assess whether the measurements from the efficacy trial and cross-sectional survey were comparable. | Methods: Statistical Analysis 1^st^ paragraph |
| Study size | 10 | G6PD data were available from 175 patients with uncomplicated malaria enrolled into the efficacy trial (18) and 999 individuals from the cross-sectional survey (19, 25). The case-control study was powered to determine a minimal difference of 0.75 U/gHb between cases and controls with a 95% two-sided confidence interval and 80% power, which required 203 cases and 203 controls. The total sample size of the three studies was 1,680 participants. | Methods: Sample Size |
| Quantitative variables | 11 | G6PD activities, measured by spectrophotometry (in U/dL), were divided by the corresponding Hb measurement (in g/dL) collected in the field to provide a result in U/gHb.  The adjusted male median was calculated for each spectrophotometry assay as described previously and defined as 100% activity (27). Following current treatment recommendations individuals with G6PD activities below 30% were categorized as G6PD deficient, those with activities between 30% and below 70% activity as G6PD intermediate, and those with activities at or above 70% as G6PD normal (5, 31, 32).  Peripheral parasitaemia was defined according to three categories: i) individuals with peripheral parasitaemia diagnosed by blood film examination or RDT and confirmed by PCR, ii) individuals with submicroscopic parasitaemia diagnosed by PCR but not microscopy or RDT, and iii) individuals without detectable peripheral parasitaemia assessed by microscopy, RDT or PCR. | Methods: Statistical Analysis, 2^nd^, 3^rd^ and 4^th^ paragraph |
| Statistical methods | 12 | Normally distributed continuous data were compared using Students t-test or paired Students t-test. Non-normally distributed continuous data were compared using the Wilcoxon signed-rank test, the Hodges Lehman estimator or the Kruskal–Wallis equality-of-populations rank test . Unpaired categorical data were compared using the Chi-square test or Fishers exact test as appropriate. For each spectrophotometry assay, a multivariable regression model was developed to predict non-normalized G6PD activity (in U/dL). Based on biological plausibility the following predictors were included in both models: Hb, sex, and G6PD genotype. In addition malaria status (negative, PCR only positive or microscopy positive) was added for the comparison of participants with and without parasitemia and history of malaria for the case control study. | Methods: Statistical Analysis, 5^th^ paragraph |
|  |  | Individuals were enrolled into three studies at the same location in the Chittagong Hill Tracts (CHT), Bangladesh: a clinical trial of patients presenting with uncomplicated P. falciparum or P. vivax malaria (18), a cross-sectional survey (19), and a case-control study. First, we determined whether G6PD activity differed between patients with symptomatic malaria, recruited from the efficacy trial, and afebrile and malaria negative participants from the cross-sectional survey. We then compared G6PD activities between participants with and without a history of malaria, all of whom were afebrile and free of malaria at the time point of G6PD measurement. | Methods: 2^nd^ paragraph |

Continued on next page

| Results | | | |
| --- | --- | --- | --- |
| Participants | 13* | - Clinical trial: 175 participants - Cross sectional survey: 999 participants - Case control study: 506 participanst   Due to the nature of this project no lost to follow up | Results: “Cross sectional survey and clinical trial”, 2^nd^ paragraph and “Case Control Study” 1^st^ paragraph |
|  |  | (b) NA |  |
|  |  | (c) Consider use of a flow diagram | Figure 2 |
| Descriptive data | 14* | (a) NA |  |
|  |  | (b) NA |  |
|  |  | (c) *NA* |  |
| Outcome data | 15* | - *Clinical trial:* 175 malaria cases by microscopy - *Cross sectional survey:* 7 malaria cases by microscopy, 36 malaria positive by PCR only - *Case control study:* 253 cases with history of malaria, 253 controls without no history of malaria | Results: “Cross sectional survey and clinical trial”, 2^nd^ paragraph and “Case Control Study” 1^st^ paragraph |
| Main results | 16 | In the cross-sectional survey and clinical trial, 15.5% (182/1174) of participants had peripheral parasitaemia detected by microscopy or RDT, 3.1% (36/1174) were positive by PCR only and 81.4% (956/1174) were aparasitaemic. Individuals without malaria had significantly lower G6PD activity (median: 6.9 U/gHb (interquartile range (IQR):5.2 to 8.6)), compared to those with peripheral parasitaemia detected by microscopy or RDT (7.9 U/gHb, IQR: 6.6 to 9.8, p<0.001), but similar to those with low-level parasitaemia detected by PCR alone (6.1 U/gHb, IQR: 4.8 to 8.6, p=0.312). In total 7.7% (14/182) patients with malaria had G6PD activity <70% compared to 25.0% (248/992) of participants with submicroscopic or no parasitemia (Odds Ratio OR=0.25 [95%CI 0.14-0.44]); p<0.0001).  In the case control study, the median G6PD activity was 10.3 U/gH (IQR: 8.8 to 12.2) in the 253 patients with a history of malaria and 10.2 U/gHb (IQR: 8.7 to 11.8) in the 253 individuals without a history of malaria (p=0.323). The proportion of individuals with less than 70% activity was 11.5% (29/253) in the cases and 15.4% (39/253) in the controls (OR=0.7 [95%CI 0.41 – 1.23]; p=0.192). | Results: “Cross sectional survey and clinical trial” 1^st^, 2^nd^, 4^th^, 5^th^ paragraph and “Case Control Study” 2^nd^ paragraph |
| Other analyses | 17 | NA |  |
| Discussion | | | |
| Key results | 18 | Our study highlights a significant difference in G6PD activity between patients with clinical malaria, and asymptomatic individuals who were either aparasitaemic or had very low-level parasitaemia that was only detected by PCR. This difference could not be accounted for by a protective effect against malaria from low G6PD activities. | Discussion: 1^st^ paragraph |
| Limitations | 19 | Our study has some important limitations. We relied on non-contemporaneous collection of specimens from a clinical trial and cross-sectional survey, rather than clinical observation of the same patient over multiple times points with and without malaria. However when comparing results from laboratory quality control testing, results did not differ significantly, suggesting that results between both studies are comparable.  In the case-control series, participants were tested using a combination RDT for both P. falciparum and P. vivax, and this is known to have lower sensitivity for identifying patients with P. vivax malaria (46). It is also possible that some participants had malaria which was not detected due to treatment-seeking outside of the surveillance system. However, patients have to pay for services and drugs provided at private clinics, pharmacies, and to some extent at public hospitals; it is unlikely that many participants would prefer this option over free treatment delivered at home as part of the study. A subset of 100 participants from the same case and control arms respectively were included in a separate study on the perceptions of malaria (47). A total of 6/100 participants from the control arm recalled having had malaria before, 1 case in contrast could not recall any previous malaria episode. These findings may have been affected by recall and response bias, however, suggest that the majority of participants was categorized correctly. | Discussion: 4^th^ and 5^th^ paragraph |
| Interpretation | 20 | In conclusion, our study highlights the potential impact of acute malaria on G6PD status, which may influence treatment allocation of an 8-aminoquinoline drug. | Discussion 8^th^ paragraph |
| Generalisability | 21 | If confirmed prospectively, 8-aminoquinoline based radical cure may be safer than assumed in patients with clinical malaria, compared to individuals who have submicroscopic parasitaemia or who are aparasitaemic. | Discussion 8^th^ paragraph |
| Other information | | | |
| Funding | 22 | This study was funded by the Bill and Melinda Gates Foundation (OPP1054404) and the Wellcome Trust (Senior Fellowship in Clinical Science awarded to RNP, 200909). SA is supported by a Georgina Sweet Award for Women in Quantitative Biomedical Science and KT is a CSL Centenary fellow. No funding bodies had any role in study design, data collection and analysis, decision to publish, or preparation of the manuscript. | TBD |

*Give information separately for cases and controls in case-control studies and, if applicable, for exposed and unexposed groups in cohort and cross-sectional studies.

**Note:** An Explanation and Elaboration article discusses each checklist item and gives methodological background and published examples of transparent reporting. The STROBE checklist is best used in conjunction with this article (freely available on the Web sites of PLoS Medicine at http://www.plosmedicine.org/, Annals of Internal Medicine at http://www.annals.org/, and Epidemiology at http://www.epidem.com/). Information on the STROBE Initiative is available at www.strobe-statement.org.
