# Supplementary material for: Glucose-6-phosphate dehydrogenase activity in individuals with and without malaria: Analysis of clinical trial, cross-sectional and case–control data from Bangladesh
Source: PLoS Med. 2021 Apr 23;18(4):e1003576. doi: 10.1371/journal.pmed.1003576 (PMC8064587; doi:10.1371/journal.pmed.1003576)
Supplement: Attachment — Submitted filename: ley.pdf [file pmed.1003576.s009.pdf]

This paper reports a set of inter–linked studies of Glucose–6–phosphate–dehydrogenase activity in a rural area of Bangladesh.

I was asked for a statistical report and I interpret that to include all aspects of the design and conduct of the study.

Points of detail

Page 9 I can see that categorising G6PD activity may be helpful for presentation but it does miss the chance to see whether these cut–offs are in fact appropriate. Categorising an essentially continuous variable wastes information (Altman and Royston, 2006; Royston et al., 2006) and leads to models which are often implausible as they predict the effect remaining flat within categories and then jumping to a new value at the category boundary. The position would of course have been worse if the choice had been made with knowledge of the data (Altman et al., 1994).

Page 10 Strictly speaking multivariable is meant here and elsewhere (Hidalgo and Goodman, 2013). Multivariate means multiple variables on the left hand side.

Page 10 I do not see a clear scientific or clinical justification put forward for doing variable selection. Selecting a subset of variables in a way driven by the data leads to a model which is unlikely to replicate (Babyak, 2004). It would be better to fit a model which relied on theory or, if the goal is just adjustment, to fit a model with all the predictors.

Page 11 For completeness I think the y–axis should be labelled.

Page 12, Table 1 I wonder whether it might be better to show the difference in means along with its confidence interval rather than confidence intervals for the individual means?

Page 15, Figure 2 We definitely do need a label for the y–axis here. The reader is going to make the assumption that all four plots are on the same scale but if these are raw frequencies then with sample sizes varying from 48 to 595 that is clearly not the case.

Page 16 What was the value of the correlation? A confidence interval might also be helpful to get some idea of precision so we can evaluate the import of its failure to meet some arbitrary level of statistical significance.

Page 17 With only 2 or 3 observations why not just give them? The IQR and range use more space to give us the same information.

Page 18 Perhaps give the unit for the continuous ones rather than just saying continuous so we can see what the estimated coefficient refers to? I am not sure why weight had to be categorised into two categories when it is inherently continuous. In general for categorical variables we could use the explicit reference category being quoted too.

Page 19, Figure 4 Label for y–axis again.

Page 20 As for page 18. This does raise the issue of why the variants displayed here are not the same ones as on page 18. Are these the totality of possible variants in that sample or are the omitted ones an artefact of using variable selection? If the latter that seems to me a further argument against variable selection.

Nice to receive something with a majority of in–country authors for a change.

Point of more substance

I assume from the use of quantiles on page 11 that a non–parametric test was used here although not the Wilcoxon signed–rank test specified in the methods since these are independent samples. It would really be helpful here to have more on the differences especially given the authors comments on page 22. Then we could see whether there is a very precise estimate of the differences. Even without that information I suspect the authors are correct that any differences are in fact not of major scientific importance. Why not use the Hodges–Lehmann estimator which does yield a confidence interval? There are other options of course.

Summary

Mostly for clarification. Relying so much on p–values rather than measures of effect size could, and I believe should, be avoided.

Michael Dewey

References

D G Altman and P Royston. The cost of dichotomising continuous variables. *British Medical Journal*, 332:1080, 2006.

D G Altman, B Lausen, W Sauerbrei, and M Schumacher. Dangers of using 'optimal' cutpoints in the evaluation of prognostic factors. *Journal of the National Cancer Institute*, 86:829–835, 1994.

M A Babyak. What you see may not be what you get: a brief, nontechnical introduction to overfitting in regression–type models. *Psychosomatic Medicine*, 66:411–421, 2004.

B Hidalgo and M Goodman. Multivariate or multivariable regression. *American Journal of Public Health*, 103:39–40, 2013.

P Royston, D G Altman, and W Sauerbrei. Dichotomizing continuous predictors in multiple regression: a bad idea. *Statistics in Medicine*, 25:127–141, 2006.
