# Supplementary material for: Glucose-6-phosphate dehydrogenase activity in individuals with and without malaria: Analysis of clinical trial, cross-sectional and case–control data from Bangladesh
Source: PLoS Med. 2021 Apr 23;18(4):e1003576. doi: 10.1371/journal.pmed.1003576 (PMC8064587; doi:10.1371/journal.pmed.1003576)
Supplement: Attachment — Submitted filename: Reviewer 3 - G6PD_Malaria_v01.1 mod 22 Nov 2020.docx [file pmed.1003576.s010.docx]

**Variability of Glucose-6-Phosphate Dehydrogenase activity in individuals with and without malaria in the Chittagong Hill Tracts of Bangladesh**

Benedikt Ley^1*^, Mohammad Shafiul Alam^2^, Mohammad Golam Kibria^2^, Jutta Marfurt^1^, Ching Swe Phru^2^, Jenifar Quaiyum Ami^2^, Kamala Thriemer^1^, Sarah Auburn^1^, Nusrat Jahan^2^, Fatema Tuj Johora^2^, Mohammad Sharif Hossain^2^, Cristian Koepfli^3^, Wasif Ali Khan^2^, Ric N. Price^1,4,5^

1. Global and Tropical Health Division, Menzies School of Health Research and Charles Darwin University, Darwin, Australia
2. Infectious Diseases Division, International Centre for Diarrhoeal Disease Research, Bangladesh (icddr,b), Dhaka 1212, Bangladesh
3. Eck Institute for Global Health, Department of Biological Sciences, University of Notre Dame, USA
4. Mahidol-Oxford Tropical Medicine Research Unit (MORU), Faculty of Tropical Medicine, Mahidol University, Bangkok, Thailand
5. Centre for Tropical Medicine and Global Health, Nuffield Department of Clinical Medicine, University of Oxford, Oxford, United Kingdom.

*Corresponding author: Benedikt.Ley@menzies.edu.au

Keywords: G6PD activity, G6PD deficiency, Bangladesh, Cross sectional Survey, *Plasmodium vivax*, Primaquine, Tafenoquine

**Abstract:**

**Background:** Glucose-6-phosphate dehydrogenase (G6PD) activity is dependent upon G6PD genotype and age of the red blood cell (RBC) population, with younger RBCs having higher activity. Peripheral parasitemia with *Plasmodium spp.* induces hemolysis, replacing older RBCs with younger cells with higher G6PD activity. This study aimed to assess whether G6PD activity varies between individuals with and without malaria or a history of malaria.

**Methods and Findings:** Individuals, with and without malaria, living in the Chittagong Hill Tracts of Bangladesh were enrolled into three complementary studies: i) a clinical efficacy trial of patients with uncomplicated malaria, ii) a cross-sectional survey, and iii) a matched case-control study of aparasitemic individuals with and without a history of malaria. G6PD activity was compared between individuals with and without malaria diagnosed by microscopy, rapid diagnostic test (RDT) or polymerase chain reaction (PCR) and also in aparasitaemic participants with and without a history of malaria. In the cross-sectional survey and clinical trial, 15.5% (182/1174) of participants had peripheral parasitaemia detected by microscopy or RDT, 3.1% (36/1174) were positive by PCR only and 81.4% (956/1174) were aparasitaemic. Individuals without malaria had significantly lower G6PD activity (median: 6.9 U/gHb (interquartile range (IQR):5.2 to 8.6)), compared to those with peripheral parasitaemia detected by microscopy or RDT (7.9 U/gHb, IQR: 6.6 to 9.8, p<0.001), but similar to those with low-level parasitaemia detected by PCR alone (6.1 U/gHb, IQR: 4.8 to 8.6, p=0.312). In total 7.7% (14/182) patients with malaria had G6PD activity <70% compared to 25.0% (248/992) of participants with submicroscopic or no parasitemia (Odds Ratio OR=0.25 [95%CI 0.14-0.44]); p<0.0001).

In the longitudinal cohort, the median G6PD activity was 10.3 U/gH (IQR: 8.8 to 12.2) in the 253 patients with a history of malaria and 10.2 U/gHb (IQR: 8.7 to 11.8) in the 253 individuals without a history of malaria (p=0.323). The proportion of individuals with less than 70% activity was 11.5% (29/253) in the cases and 15.4% (39/253) in the controls (OR=0.7 [95%CI 0.41 – 1.23]; p=0.192). Limitations of the study included the non-contemporaneous nature of clinical trials and cross-sectional surveys.

**Conclusions:** Patients with acute malaria had significantly higher G6PD activity than individuals without malaria and this could not be accounted for by a protective effect of G6PD deficiency. These findings suggest that G6PD deficient patients with malaria may have higher than expected G6PD enzyme activity and an attenuated risk of primaquine-induced haemolysis compared to the risk when not infected.

Primary funding for the study was through the Bill and Melinda Gates Foundation, The Wellcome Trust, and the Australian National Health and Medical Research Council.

**Author Summary**

*Why was this study done?*

Primaquine and tafenoquine are the only drugs that prevent relapses of the vivax malaria parasite. Both drugs cause significant side-effects in patients with low activities of the G6PD enzyme. Approximately 400 million people worldwide are affected by low G6PD activities.

*What did the researchers do and find?*

We measured G6PD activities among individuals with and without malaria and found G6PD activities to be significantly higher among malaria patients. We then enrolled healthy individuals with and without a history of malaria to assess whether people with low G6PD activities were less likely to contract malaria; G6PD activities did not differ significantly. We conclude that G6PD activity increases during a malaria infection and the observed clinically relevant difference is not due to people with lower G6PD activities being less likely to contract malaria.

*What do these findings mean?*

These findings suggest that standard treatment for vivax malaria may be safer than assumed, however these findings will need to be confirmed.

**Introduction**

Over the last two decades, significant progress has been made in reducing the burden of malaria. However, the impact of intense malaria control activities has been far greater for *Plasmodium falciparum* than *P. vivax*, which is now the predominant malaria species in many co-endemic countries (1, 2). Unlike *P. falciparum, P. vivax* forms dormant liver stages (hypnozoites) that can reactivate weeks to months after the first infection, causing recurrent episodes of febrile illness associated with significant morbidity and mortality (3). To date, the only drugs that can kill hypnozoites are the 8-aminoquinolines compounds, primaquine (PQ), and tafenoquine (TQ), which although generally well tolerated, can cause severe hemolysis in individuals with glucose-6-phosphate dehydrogenase (G6PD) deficiency (4). In view of the risk of severe drug-induced hemolysis, the World Health Organization (WHO) recommends that all patients should be tested for G6PD deficiency (G6PDd) prior to prescribing PQ or TQ (5).

G6PDd is an inherited X-linked enzymopathy with highest prevalence in malaria endemic settings (6, 7). The strong geographic overlap between current and historic areas of malaria transmission and G6PDd suggest an interaction between these exposures, however investigations of this interaction are conflicting (8). The correlation between G6PD genotype and phenotype is poor (9-12), since G6PD activity is also determined by a range of factors including the age of the red blood cell (RBC) population, with younger RBCs exhibiting higher levels of G6PD activity compared to older ones, and, in heterozygous females, the degree of lyonization (13).

In the presence of oxidative stress, triggered by certain foods, drugs, and infection, G6PD deficient RBCs hemolyse to a far greater degree compared to G6PD normal RBCs (14). Plasmodia are intraerythrocytic parasites that induce intravascular hemolysis, and this occurs in both parasitized and unparasitized red blood cells (15, 16). Whilst *P. vivax* preferentially invade younger RBCs, hemolysis of non-parasitized cells may be particularly marked in older RBCs with lower G6PD activities (17). We hypothesized that G6PD activity is elevated in patients with malaria compared to individuals without malaria, attributable to acute parasitaemia, and that this enzyme elevation occurs to a greater degree than that attributable to inherent protection from infection in these individuals. The aim of this study was to determine whether G6PD activity varies in individuals with and without malaria and whether this was attributable to a reduced risk of malaria.

**Methods**

Individuals were enrolled into three studies at the same location in the Chittagong Hill Tracts (CHT), Bangladesh: a clinical trial of patients presenting with uncomplicated *P. falciparum* or *P. vivax* malaria (18), a cross-sectional survey (Ley et al, accepted for publication in PLoS NTD), and a case-control study. First, we determined whether G6PD activity differed between patients with symptomatic malaria, recruited from the efficacy trial, and afebrile and malaria negative participants from the cross-sectional survey. We then compared G6PD activities between participants with and without a history of malaria, all of whom were afebrile and free of malaria at the time point of G6PD measurement.

*Clinical Trial (18)*

The clinical trial was conducted between September 2014 and February 2015 in Alikadam in the district of Bandarban (CHT). Patients with uncomplicated malaria diagnosed by microscopy were enrolled and treated according to national treatment guidelines. Venous blood samples were collected for phenotypic and genotypic G6PD testing.

*Cross-sectional survey (19) (*Ley et al, accepted for publication in PLoS NTD*)*

The cross-sectional survey was conducted in the area around Alikadam, district of Bandarban, between August 2015 and January 2016 to assess the prevalence of G6PDd in the local population. Venous blood, collected from one household member of randomly selected households, was analyzed for peripheral parasitaemia and quantified by microscopy and malaria rapid diagnostic (FalciVax, Zephyr Biomedicals, Goa, India). Parasite species was confirmed by PCR (Ley et al, accepted for publication in PLoS NTD). G6PD status was determined phenotypically and genotypically as described below.

*Case-control study*

In 2009, a prospective demographic surveillance system (DSS) was established in the Bandarban district amongst a population of 20,558 and is currently part of the INDPETH network (20-22). Coordinates of every household within the surveillance area were recorded and all households were visited between 2009 and 2010 to enroll participants (23). Participants were visited every three months between 2009 to 2014, every four months in 2015, and since 2016 household visits are conducted bi-annually. At the end of 2018, 19,117 people were under surveillance. All participants were assigned a unique identifier (ID) at the initial visit and a history of malaria was recorded, at follow-up visits, in- and outmigration, birth, deaths, and change of address were recorded. Febrile participants were asked to notify the study clinical staff for a malaria rapid diagnostic test (RDT, FalciVax, Zephyr Biomedicals, Goa, India). Participants who were RDT positive were provided with free antimalarial treatment according to national treatment guidelines. The episode of malaria was recorded, and the participant was revisited at the end of the provided treatment course.

This cohort was used to generate a case-control study comprising of individuals, who were all afebrile and aparasitemic at the time of enrolment. Cases were selected randomly from DSS participants who had had at least one episode of malaria confirmed by microscopy during their monitoring within the DSS. Controls were selected from participants with no recorded episodes of malaria during their monitoring within the DSS. Cases and controls were selected in a ratio of 1:1, and matched according to the duration under surveillance, sex, age, and village of residence.

The recorded history of malaria was confirmed by all participants before enrolment and the absence of parasitemia was determined by RDT (*CareStart*™ Malaria HRP2/pLDH (Pf/Pv) Combo RDT, ACCESS BIO INC, NJ, USA ) and confirmed by microscopic blood film examination. Thick and thin microscopy films were stained with Giemsa and parasites counted per 200 white blood cells or 2000 red blood cells by two independent readers and the mean of both readings was recorded. In the case of discrepant results, a third reading was done by an expert microscopists and this finding was considered final.

After informed written consent, individuals were included in the study if no peripheral parasitaemia was detected by either RDT or microscopy, they had an axillary temperature of less than 37.5°C, and no history of fever in the preceding 48 hours. Individuals not fulfilling these criteria were excluded and replaced by participants fitting all criteria until the desired sample size was reached. A venous blood sample was collected from all participants to measure hemoglobin (Hb) concentration, G6PD activity by spectrophotometry and genotyping of the *G6PD* variant.

*Hemoglobin (Hb) measurement and G6PD phenotypic testing*

Hb concentration was measured immediately after venipuncture, using a Hemocue 301 (Angelholm, Sweden). Venous samples were stored at 4°C and a cold chain was maintained until samples reached the reference laboratory at the International Center for Diarrheal Disease Research in Bangladesh (icddr,b) in the capital Dhaka.

G6PD activity was measured using a UV-1800 Shimadzu (Shimadzu, Kyoto, Japan). Samples from the efficacy trial and the cross-sectional survey were tested using kits and controls (normal and deficient) from Randox (Crumlin, UK) at 37°C (18, 24). Samples from the case-control study were analyzed at 30°C using kits from Pointe Scientific (Canton, USA), quality control was done using normal, intermediate, and deficient controls from ACS Inc. (Fishers, USA) (18, 19). Controls were run daily before testing which was only commenced if the control readings were within the recommended G6PD activity range. All measurements were run in duplicate and repeated if the difference between the first and second measurement was greater than 10%.

*G6PD Genotypic Testing*

All samples from patients with less than 70% G6PD activity of the adjusted male median (AMM) (25) and a randomly selected subset of samples with higher G6PD activities were genotyped by PCR at a reference laboratory in Dhaka for the following variants: Mahidol, Viangchan, Mediterranean and Orissa as described previously (26). All PCR products were also sequenced to identify the Kalyan-Kerala variant (27) (Ley et al, accepted for publication in PLoS NTD).

*Sample Size*

G6PD data were available from 175 patients with uncomplicated malaria enrolled into the efficacy trial (18) and 999 individuals from the cross-sectional survey (24) (Ley et al, accepted for publication in PLoS NTD). The case-control study was powered to determine a minimal difference of 0.75 U/gHb between cases and controls with a 95% two-sided confidence interval and 80% power, which required 203 cases and 203 controls. The total sample size of the three studies was 1,680 participants.

*Statistical analysis*

Clinical and laboratory data were entered on paper-based case report forms (CRFs) and digitalized using Epidata version 3.1 (EpiData, Odense, Denmark) (28). All analyses were done using Stata version 14 (Stata Corp, College Station, USA).

G6PD activities, measured by spectrophotometry (in U/dL), were divided by the corresponding Hb measurement (in g/dL) collected in the field to provide a result in U/gHb. The results from Randox control samples generated from the clinical and survey participants were compared to assess whether the measurements from the efficacy trial and cross-sectional survey were comparable.

The adjusted male median was calculated for each spectrophotometry assay as described previously and defined as 100% activity (25). Individuals with G6PD activities below 30% were categorized as G6PD deficient, those with activities between 30% and below 70% activity as G6PD intermediate, and those with activities at or above 70% as G6PD normal (29).

Peripheral parasitaemia was defined according to three categories: i) individuals with peripheral parasitaemia diagnosed by blood film examination or RDT and confirmed by PCR, ii) individuals with submicroscopic parasitaemia diagnosed by PCR but not microscopy or RDT, and iii) individuals without detectable peripheral parasitaemia assessed by microscopy, RDT or PCR.

Normally distributed continuous data were compared using Students t-test or paired Students t-test. Non-normally distributed continuous data were compared using the Wilcoxon signed-rank test or the Kruskal–Wallis one-way analysis of variance. Unpaired categorical data were compared using the Chi-square test or Fishers exact test as appropriate. For each spectrophotometry assay, a multivariate regression model was developed to predict non-normalized G6PD activity (in U/dL) using a backward selection of variables. Non-normalized G6PD activity was used for the dependent variable and Hb concentration included in the model as an independent covariate. Independent variables initially included were Hb, sex, age, weight, body temperature, ethnicity (indigenous or Bengali), G6PD genotype, and malaria status. In the case-control study the following additional variables were also assessed: the time between sample collection and spectrophotometry, self-reported fever in the last 48 hours, self-reported fever in the last 30 days, malaria in the last 6 months, any blood transfusion in the last 6 months, number of malaria episodes since being included in the DSS, last diagnosed malaria species and corresponding malaria parasite count at diagnosis.

*Ethics*

All protocols were approved by the Ethics Review Committee of the icddr,b, Bangladesh and the Human Research Ethics Committee of the Northern Territory Department of Health and Menzies School of Health Research, Australia. Protocol specific identification numbers together with the exact title of the protocol can be found in the supplement (S1 Table). Written informed consent was collected from all participants before enrolment and in addition, written assent was collected from all minors above the age of 11 years.

**Results**

*Cross sectional survey and clinical trial*

Quality control samples were run on 75 normal and deficient control isolates during the efficacy trial and 255 control isolates during the cross-sectional survey. The median reading from normal controls in the efficacy trial was 8.7U/gHb (inter quartile range (IQR): 8.2 – 9.2) compared to 8.3 U/gHb (IQR: 7.8 – 8.7) in the cross-sectional survey (median: 8.3U/gHb, p<0.001). The median reading from deficient controls was 1.3 U/gHb (IQR: 1.0 – 1.7) in the efficacy trial, and 1.6U/gHb (IQR: 1.4 – 1.8) in the cross-sectional survey (p<0.001) (Fig 1).

Figure 1: Distribution of G6PD activity in normal and deficient controls per study

A total of 1,174 participants were enrolled in the clinical trial and cross-sectional survey and assessed by spectrophotometry, of whom 182 (15.5%) had patent parasitaemia, 36 (3.0%) sub-patent parasitaemia, and 956 (81.4%) were aparasitemic (Fig 2).

**Figure 2: Study profile of participants enrolled in the treatment efficacy trial and cross-sectional survey**

**4 participants had a P. falciparum parasitaemia detected by RDT, but not by microscopy*

Overall, 25.2% (134/531) of males had microscopic parasitaemia, compared to 7.5% (48/643) of females; p<0.001. All subsequent analyses were therefore stratified per sex. Participants in the treatment efficacy study differed significantly from those in the survey, and were younger, had lower body weight, were more likely to be febrile, and had lower Hb concentration (Table 1).

**Table 1: Baseline variables stratified by study, microscopic malaria and submicroscopic malaria for participants of the treatment efficacy trail and cross-sectional survey.**

| **Comparing studies** | | | |
| --- | --- | --- | --- |
| **Variable** | **Clinical Trial (n=175)** | **Cross Sectional Survey (n=999)** | **P** |
| **Females (%)** | 45 (25.7) | 598 (59.9) | **<0.001** |
| **Mean age**  **years (95%CI)** | 22.0 (20.1 – 23.9) | 32.5 (31.4 – 33.6) | **<0.001** |
| **Mean body weight kg (95%CI)** | 41.4 (39.1 – 43.7) | 44.3 (43.6 – 45.0) | **0.002** |
| **Mean body temperature**  **°C (95%CI)** | 37.8 (37.6 – 37.9) | 36.2 (36.2 – 36.3) | **<0.001** |
| **Mean Hb concentration**  **g/dL (95%CI)** | 12.6 (12.3 – 12.9) | 13.0 (12.9 – 13.1) | **0.002** |
| **n with Hb<7g/dL** | 0 | 6 | 0.379 |
| **Geometric mean Parasitaemia µl^-1^ (95%CI, n)** | - *P. falciparum:* 5,692 (3,745 – 8,651, 111) - *P. vivax:* 2,661 (1,675 – 4,228, 54) - *P. falciparum* + *P. vivax* mixed: 9,978 (1,279 – 77,844, 10) | - *P. falciparum:* 3,986 (77 – 206,902, 3) | - |
| **Comparing participants with parasitemia confirmed by microscopy vs. participants with no parasitemia** | | | |
| **Variable** | **Microscopic parasitemia (n=182)** | **No parasitemia (n=956)** | - |
| **Females (%)** | 48 (26.4) | 579 (60.6%) | **<0.001** |
| **Mean age**  **years (95%CI)** | 21.6 (19.8 – 23.5) | 32.8 (31.7 – 33.9) | **<0.001** |
| **Mean body weight**  **kg (95%CI)** | 41.0 (38.7 – 43.3) | 44.5 (43.8 – 45.3) | **<0.001** |
| **Mean body temperature**  **°C (95%CI)** | 37.7 (37.6 – 37.9) | 36.2 (36.2 – 36.3) | **<0.001** |
| **Mean Hb concentration**  **g/dL (95%CI)** | 12.6 (12.3 – 12.9) | 13.0 (12.9 – 13.1) | **<0.001** |
| **Severe anaemia Hb<7g/dL n(%)** | 0 | 6 (0.6%) | 0.597 |
| **Geometric mean Parasitaemia µl^-1^**  **(95%CI)** | - *P. falciparum:* 5.639 (3,744 – 8,492, 114) - *P. vivax:* 2,661 (1,675 – 4,228, 54) - *P. falciparum* + *P. vivax* mixed: 9,978 (1,279 – 77,843, 10) | - | - |
| **Comparing participants with parasitemia confirmed by PCR vs. participants with no parasitemia** | | | |
| **Variable** | **PCR positive only (n=36)** | **No parasitemia (n=956)** | - |
| **Females (%)** | 16 (44.4) | 579 (60.6%) | 0.053 |
| **Mean age**  **years (95%CI)** | 29.6 (23.7 – 35.5) | 32.8 (31.7 – 33.9) | 0.145 |
| **Mean body weight**  **kg (95%CI)** | 41.7 (37.3 – 46.1)) | 44.5 (43.8 – 45.3) | 0.080 |
| **Mean body temperature**  **°C (95%CI)** | 36.4 (36.3 – 36.6) | 36.2 (36.2 – 36.3) | 0.012 |
| **Mean Hb concentration**  **g/dL (95%CI)** | 13.1 (12.7 – 13.5) | 13.0 (12.9 – 13.1) | 0.354 |
| **n with Hb<7g/dL** | 0 | 6 | 0.801 |

Individuals with microscopic parasitaemia had a median G6PD activity of 7.86 U/gHb (IQR: 6.62-9.84) compared to 6.88 U/gHb (IQR: 5.16-8.59) in aparasitemic participants, p<0.001. Conversely there was no significant difference in G6PD activity between submicroscopic and aparasitemic individuals; p>0.05 (Table 2, Fig. 3 and S1 Fig).

**Table 2: Distribution of median G6PD activities stratified by sex and malaria status**

|  | **Males (Median G6PD activity in U/gHb (IQR)** | **Females (Median G6PD activity in U/gHb (IQR)** | **All (Median G6PD activity in U/gHb (IQR)** |
| --- | --- | --- | --- |
| **Aparasitaemic**  **(Reference)** | 6.67 (4.94 – 8.54)  n=377) | 7.01 (5.36 – 8.67)  n=579) | 6.88 (5.16 – 8.59, 956) |
| **Subpatent parasitaemia** | 6.84 (4.35 – 9.45)  n=20, p=0.975 | 6.08 (4.92 – 6.87)  n=16, p=0.146 | 6.09 (4.79 – 8.62)  n=36, p=0.312 |
| **Microscopic parasitemia** | 7.82 (6.59 – 10.00)  n=134, p<0.001 | 8.06 (6.89 – 9.53)  n=48, p<0.001 | 7.86 (6.62 – 9.84)  n=182, p<0.001) |

*IQR: interquartile range, n=number of participants*

**significant difference compared to aparasitemic reference*

**Figure 3: G6PD activity (in U/gHb) distribution stratified by microscopic parasitaemia and sex in the efficacy trial and cross-sectional survey**

*Vertical lines indicate 30%, 70%, and 100% G6PD activity of the adjusted male median (from left to right). 100% G6PD activity = 7.4U/gHb. Participants with submicroscopic parasitemia included in malaria negative cohort.*

The AMM was defined as 7.4 U/gHb (IQR: 5.68 – 9.07), based on 496 normal males. A total of 77.7% (912/1,174) of participants were categorized as G6PD normal (>70%), 14.3% (168/1,174) had intermediate G6PD activity (30-70%) and 8.0% (94/1,174) were G6PD deficient (<30%).

There was no significant difference in G6PD status between aparasitaemic and subpatent parasitaemic individuals (Table 2), and these groups were therefore pooled (Table 3). Overall, 9.2% (92/992) of those without microscopic parasitaemia had G6PD activities below 30% compared to 1.1% (2/182) of individuals with microscopic parasitaemia; (Odds Ratio (OR)=9.2, 95%CI: 2.43 to 77.71, p<0.0001). At the 70% G6PD threshold, 33.3% (244/992) of participants without microscopic parasitemia were G6PD deficient compared to 7.6% (14/182) of patients with microscopic parasitaemia (OR= 4.0, 95%CI: 2.26 to 7.61, p<0.001) (Table 2). This difference was particularly apparent in females, with 23.0% (137/595) of those with subpatent parasitaemia or aparasitaemia being intermediate or severely G6PD deficient compared to 2.1% (1/48) of those with malaria (OR= 14.1 (95%CI: 2.4 to 570.9)); p<0.001.

In the 178 individuals with microscopic parasitaemia the geometric mean parasitaemia was 5,107 (95%CI 3,632 – 7,180) parasites µl^-1^. There was no significant correlation between peripheral parasitemia and G6PD activity (p=0.991), and this did not change when stratifying by species (all p>0.05).

*Table 3: Proportions of phenotypic G6PD deficient, intermediate and normal participants among individuals with no parasitemia, parasitemia confirmed by PCR only and confirmed by microscopy*

|  | **Aparasitemia**  **n (%)** | **Subpatent parasitaemia**  **n (%)** | **Microscopic parasitaemia**  **n (%)** | **Total**  **n (%)** |
| --- | --- | --- | --- | --- |
| **G6PD deficient** | 88 (9.2) | 4 (11.1) | 2 (1.1) | 94 (8.0) |
| **Intermediate Deficient** | 149 (15.6) | 7 (19.4) | 12 (6.6) | 168 (14.3) |
| **G6PD normal (reference)** | 719 (75.2) | 25 (69.4) | 168 (92.3) | 912 (77.7) |
| **Total** | 956 (100.0) | 36 (100.0) | 182 (100.0) | 1,174 |

Genotyping was undertaken in 29.6% (348/1,174) of patients, including all 94 patients with less than 30% G6PD activity, 79.8% (134/168) of randomly selected patients with intermediate G6PD activity , and 13.2% (120/912) of randomly selected G6PD normal patients. A known *G6PD* variant was identified in 31.6% (110/348) of samples, variants showed a considerable range of G6PD activities (Table 4 and S2 Table)

**Table 4: G6PD variants and corresponding G6PD activities in participants of the Clinical trial and cross- sectional survey**

| **G6PD variant** | **Malaria** | | **No malaria** | | **p** |
| --- | --- | --- | --- | --- | --- |
|  | **n (% per column)** | **Median G6PD activity in U/gHb (IQR, min - max)** | **n (% per column)** | **Median G6PD activity in U/gHb (IQR, min-max)** |  |
| **Microscopic** | | | | | |
| **Mahidol homozygous** | 2 (40.0) | 2.03  IQR: 0.02 – 4.03  Range: 0.02-4.03) | 56 (53.3) | 0.51 (0.16 – 0.78, 0.00 – 13.06) | 0.448 |
| **Mahidol heterozygous** | 1 (20.0) | 5.38 (n=1) | 42 (40.0) | 3.72 (2.06 – 4.85, 0.0 – 14.52) | 0.348 |
| **Orissa homozygous** | 1 (20.0) | 0.7 (n=1) | 6 (5.7) | 2.77 (1.21-3.17, 0.27 – 4.19) | 0.285 |
| **Orissa heterozygous** | 1 (20.0) | 6.22 (n=1) | 0 (0.0) | n=0 | 0.045 |
| **Kalyan-Kerala homozygous** | 0 (0.0) | n=0 | 1 (1.0) | 2.07 (n=1) | 0.955 |
| **Total** | 5 (100.0) | - | 105 (100.0) | - | - |
| **Submicroscopic** | | | | | |
| **Mahidol homozygous** | 3 (60.0) | 0.19 (0.00 – 1.53, 0.00-1.53) | 53 (53.0) | 0.53 (0.18 – 0.77, 0.00 – 13.06) | 1.00 |
| **Mahidol heterozygous** | 2 (40.0) | 2.59 (2.06 – 3.11, 2.06 – 3.11) | 40 (40.0) | 3.81 (2.09 – 4.96, 0.00 – 15.52) | 1.00 |
| **Orissa homozygous** | 0 (0.0) | - | 6 (6.0) | 2.77 (1.21 – 3.17, 0.27 – 4.19) | 1.00 |
| **Kalyan-Kerala homozygous** | 0 (0.0) | - | 1 (1.0) | 2.07 (n=1) | 1.00 |
| **Total** | 5 | - | 100 | - | - |

The median non-normalized G6PD activity was 90.8 U/dL (IQR: 72.4 – 111.5). In a multivariate regression model including all 348 participants who were genotyped, the following independent factors were correlated negatively with G6PD activity: G6PD variant, female sex, and Hb concentration. Being positive for either species by microscopy and weight, but not submicroscopic parasite carriage, were correlated positively with G6PD enzyme activity (Table 5).

**Table 5: Host and parasite factors associated with non-normalized G6PD activity among participants with known G6PD variant**

| **Variable (type*)** | **β (95% CI)** | **p** |
| --- | --- | --- |
| **Contributors lowering G6PD activity** | | |
| Mahidol hemi / homozygous (cat) | -63.5 (-72.7 to -54.4) | **<0.001** |
| Orissa hemi/homozygous (cat) | -46.0 (-69.0 to -23.0) | **<0.001** |
| Mahidol heterozygous (cat) | -15.3 (-25.8 to -4.7) | **0.005** |
| Sex (females compared to males) (cat) | -15.4 (-23.1 to -7.7) | **<0.001** |
| Hb (cont) | -2.5 (-4.6 to -0.5) | **0.017** |
| **Contributors increasing G6PD activity** | | |
| Microscopic parasitemia (cat) | 22.0 (9.3 to 34.8) | **0.001** |
| Weight (cat) | 0.3 (0.0 – 0.6) | **0.038** |

*Model details: F(10, 337 = 22.96, p<0.001, adjusted R^2^ = 0.3875)*

**cat = categorical, cont= continuous*

*Case-Control Study*

A total of 506 participants were enrolled in the case-control study, 253 in each arm, which were well matched (Table 6). Before enrolment, cases had been under observation for a median time of 8.7 years (IQR: 8.4 – 8.9, range: 1.3 – 9.5) and controls for a median time of 8.6 years (IQR: 8.5 – 8.9, range: 1.9 – 9.5); p=0.669.

**Table 6: Baseline characteristics between individuals in the Case-Control study**

| **Variable** | **Cases** | **Controls** | **p** |
| --- | --- | --- | --- |
| **Females (%)** | 110 (43.5) | 110 (43.5) | 1.000 |
| **Mean age in years (95%CI)** | 33.2 (31.1 – 35.4) | 33.2 (31.1 – 35.4) | 0.988 |
| **N<5 years** | 0 | 0 | 0.917 |
| **N=5-15 years** | 60 | 62 |  |
| **N>15 years** | 193 | 191 |  |
| **Mean body weight**  **kg (95%CI)** | 45.4 (43.9 – 47.0) | 45.6 (44.0 – 47.3) | 0.850 |
| **Mean body height**  **cm (95%CI)** | 152.1 (150.5 – 153.7) | 151.7 (150.0 – 153.4) | 0.738 |
| **Mean body temperature**  **°C (95%CI, range)** | 36.0 (35.9 – 36.0) | 36.0 (35.9 – 36.0) | 0.688 |
| **Mean Hb concentration**  **g/dL (95%CI)** | 14.3 (14.0 – 14.6) | 14.4 (14.2 – 14.7) | 0.622 |
| **Severe anaemia (Hb<7g/dL)**  **N (%)** | 3 (2.7%) | 0 | 0.249 |
| **Median person years of observation (IQR, range)** | 8.67 (8.44 – 8.88, 1.33 – 9.50) | 8.62 (8.45 – 8.88, 1.91 – 9.50) | 0.669 |

The median G6PD activity was 10.2 U/gHb (IQR: 8.7 to 11.8) for cases and 10.3 U/gHb (IQR: 8.8 to 12.2) for controls; p=0.323 (Fig 4). The combined AMM for cases and controls was 10.7 U/gHb (IQR: 9.0 – 12.4). There was no significant difference between cases and controls in the proportion of patients categorized as G6PD normal, intermediate, or deficient (p=0.328), and there was no difference in participants categorized above or below the 30% threshold activity (OR=0.60, 95%CI: 0.27 to 1.29, p=0.155) or the 70% threshold activity (OR=0.71, 95%CI: 0.41 to 1.23, p=0.241); Fig 4 and Table 6.

**Figure 4: Distribution of G6PD activity stratified by gender in cases and controls**

*Vertical lines indicate 30%, 70%, and 100% G6PD activity of the adjusted male median.*

**Table 6: Distribution of G6PD deficient, intermediate, and normal participants among cases and controls**

| **G6PD category** | **Cases**  **n (%)** | **Controls**  **n (%)** | **Total** |
| --- | --- | --- | --- |
| **Deficient (<30% activity)** | 13 (5.1) | 21 (8.3) | 34 (6.7) |
| **Intermediate activity** | 16 (6.3) | 18 (7.1) | 34 (6.7) |
| **Normal Activity** | 224 (88.5) | 214 (84.6) | 438 (86.6) |
| **Total** | 253 | 253 | 506 |

All but one of the G6PD deficient (<30% activity) participant were genotyped (n=33) and a known variant identified in 97.0% (32/33); also all 34 G6PD intermediate participants were genotyped and a variant was identified in 23.5% (8/34) of participants (S3 Table).

The median non-normalized G6PD activity was 147.4 U/dL (IQR: 123.0 – 171.5). In a multivariable regression model (F(8, 59 = 34.57, p<0.001, adjusted R^2^ = 0.8003, n=68) variant genotype and being a member of one of the local ethnicities was correlated negatively with non-normalized G6PD activity. A history of fever in the last 30 days, higher body temperature, and higher Hb concentrations were correlated positively with G6PD activity, but history of malaria was not a significant predictor; p=0.682 (Table 7).

**Table 7: Significant predictors of non-normalized G6PD activity among cases and controls**

| **Variable (type*)** | **β (95% CI)** | **p** |
| --- | --- | --- |
| **Contributors lowering G6PD activity** | | |
| Mahidol hemi / homozygous (cat) | -71.0 (-81.0 to -61.0) | **<0.001** |
| Mediterranean heterozygous (cat) | 68.2 (-107.5 to -28.9) | **0.001** |
| Orissa hemi/homozygous (cat) | -29.4 (-51.7 to -7.1) | **0.011** |
| Ethnicity (indigenous) (cat) | -20.2 (-37.9 to -2.5) | **0.025** |
| **Contributors increasing G6PD activity** | | |
| Febrile illness in last 30 days (cat) | 29.2 (7.6 to 50.7) | **0.009** |
| Body temperature (cont) | 14.1 (0.1 – 28.0) | **0.048** |
| Hb concentration (cont) | 2.6 (0.6 – 4.6) | **0.012** |

*(F(8, 59 = 34.57, p<0.001, adjusted R^2^ = 0.8003, n=68)*

**cat = categorical variable, cont = continuous variable*

**Discussion**

Our study highlights a significant difference in G6PD activity between patients with clinical malaria, and asymptomatic individuals who were either aparasitaemic or had very low-level parasitaemia that was only detected by PCR. This difference could not be accounted for by a protective effect against malaria from low G6PD activities (8). Although individuals with a history of malaria were less likely to be G6PD deficient at the 30% and 70% threshold, the odds was only 0.6 and 0.7 and did not reach statistical significance.

Within an individual G6PD activity varies with the age of the RBC population, with younger RBCs, reticulocytes, and nucleated normoblasts having higher G6PD activity (13, 30-32). Malaria induces hemolysis, to which the host responds through reactive erythropoiesis, increasing the number of reticulocytes and, in severe cases, normoblasts within the bloodstream (32-35). As a consequence, the mean age of the host RBC population decreases while the mean G6PD activity increases. It is therefore plausible that G6PD activity rises during or shortly after an episode of acute malaria (36).

The WHO malaria treatment guidelines recommend that patients with malaria who have G6PD enzyme activity less than 30% should not be treated with a standard 14-day regimen of PQ, but rather PQ should be administered weekly for 8 weeks (37). These guidelines were formulated following studies suggesting that, except for the Mediterranean variant, PQ-induced hemolysis is self-limiting and that an initial dose of PQ would lead to a replacement of older RBCs with younger cells, conferring transitional low-grade resistance against PQ-induced hemolysis (29, 36, 38-41). Acute malaria may have a similar effect.

The haemolytic risk associated with PQ rises as G6PD activity falls (41). G6PD deficient individuals with activities below 30% can be identified with qualitative diagnostics that can be used in remote settings (42). Heterozygous females with intermediate G6PD activity (30% to 70%) are also at a significant risk of PQ induced hemolysis, but their diagnosis requires a quantitative enzyme assay (43). Our analysis highlights that the difference in G6PD status was most apparent in females, in whom the odds of having G6PD activities below 70% was 14 times higher among those with no microscopic parasitemia, compared to females with microscopic malaria infection. If confirmed, our findings suggest that the risk of PQ induced haemolysis in heterozygous females with malaria would be considerably less than that predicted by population-based surveys of healthy populations.

Our study has some important limitations. We relied on non-contemporaneous collection of specimens from a clinical trial and cross-sectional survey, rather than clinical observation of the same patient over multiple times points with and without malaria. During laboratory quality control testing, there was a significant difference in the enzyme activity of controls between the efficacy study and the cross-sectional survey that may have influenced the direct comparison of results. However, the absolute differences were low (less than 0.4U/gHb) and the distribution of control readings did not differ. Conversely, the difference in G6PD activities between participants with and without malaria was greater than 1U/gHb, and thus likely to reflect a true difference.

In the case-control series, participants were tested using a combination RDT for both *P. falciparum* and *P. vivax*, and this is known to have lower sensitivity for identifying patients with *P. vivax* malaria (44). It is also possible that some participants had malaria which was not detected due to treatment-seeking outside of the surveillance system. However, patients have to pay for services and drugs provided at private clinics, pharmacies, and to some extent at public hospitals; it is unlikely that many participants would prefer this option over free treatment delivered at home as part of the study. There was a trend for individuals without a history of malaria to be G6PD intermediate or severely deficient, as has been observed by others, but in our analysis this was modest (odds less than 0.7) and did not reach statistical significance (8, 45-47). If a protective effect of G6PDd against malaria was present it is likely to be a minor contributor to G6PD status and would not account for asymptomatic and parasite-free individuals being 4 to 9-fold more likely to be diagnosed as G6PD deficient in the clinical trial and cross-sectional survey.

More than 30% of participants were genotyped against a panel of variants known to be prevalent in the study area (4, 48, 49). However, a genetic variant could not be identified in a large proportion of phenotypically G6PD deficient individuals, perhaps indicative of the genetic diversity in the area (Ley et al, accepted for publication in PLoS NTD). Only patients with known variants were included in the regression model, and accordingly, these were restricted to 25% of all study participants, which could have affected their validity. Reassuringly, in the multivariate models, the phenotypic observations highlighted that malaria was a significant predictor of phenotypic G6PD activity, whereas the previous history of malaria was not.

In conclusion, our study highlights the potential impact of acute malaria on G6PD status, which may influence treatment allocation of an 8-aminoquinoline drug. If confirmed prospectively, 8-aminoquinoline based radical cure may be safer than assumed in patients with clinical malaria, compared to individuals who have submicroscopic parasitaemia or who are aparasitaemic.

**Acknowledgments:** We would like to thank all participants who agreed to participate, and all staff involved for making this work happen. icddr,b is grateful to the Governments of Bangladesh, Canada, Sweden, and the UK for providing core/unrestricted support.

**Funding:** This study was funded by the Bill and Melinda Gates Foundation (OPP1054404) and the Wellcome Trust (Senior Fellowship in Clinical Science awarded to RNP, 200909). SA is supported by a Georgina Sweet Award for Women in Quantitative Biomedical Science and KT is a CSL Centenary fellow. No funding bodies had any role in study design, data collection and analysis, decision to publish, or preparation of the manuscript.

**Supplementary Figures**

**S1 Fig: Distribution of G6PD activity (in U/gHb) in patients with and without peripheral parasitaemia**

*Vertical lines indicate 30%, 70% and 100% G6PD activity of the adjusted male median. 100% G6PD activity = 7.4U/gHb*

**Supplementary tables**

**S1 Table: Protocol names and ethics identifiers**

| **Study** | **Protocol name** | **HREC number (Australia)** | **ERC number (Bangladesh)** |
| --- | --- | --- | --- |
| Efficacy trial | A study to assess primaquine treatment guidelines on malaria in South-East Bangladesh | 2014-2228 | PR-14053 |
| Cross-sectional survey | Prevalence of G6PD deficiency and *G6PD* variants among the indigenous population of the Bandarban District, Chittagong Hill Tracts, Bangladesh (GPAT) | 2015-2336 | PR-15021 |
| Case-control study | Community and facility assessment to determine populations at risk of malaria and primaquine induced haemolysis (ACROSS) | 2017-3010 | PR-18001 |

**S2 Table: Distribution of G6PD activity among genotyped participants**

| **Variant** | **n (%)** | **Median G6PD activity (IQR, range)** |
| --- | --- | --- |
| Mahidol hemi/homozygous | 58 (16.7) | 0.51  IQR: 0.14 – 0.78  Range: 0.00 – 13.06 |
| Mahidol heterozygous | 43 (12.4) | 3.78  IQR: 2.06 – 5.07  Range: 0.00 – 14.52 |
| Orissa hemi/homozygous | 7 (2.0) | 2.69  IQR: 0.70 – 3.17  Range: 0.27 – 4.19 |
| Orissa heterozygous | 1 (0.3) | 6.22 |
| Kaylan – Kerala hemizygous | 1 (0.3) | 2.07 |
| No variant | 238 (68.4) | 4.76  IQR: 3.68 – 6.88)  Range: 0.00 – 16.53 |
| Total | 348 (100.0) | 4.19  IQR: 2.04 – 6.17)  Range: 0.00 – 16.53 |

**S3 Table: Distribution of G6PD variants among cases and controls and corresponding G6PD activities**

| **G6PD variant** | **Cases** | | **Control** | |  |
| --- | --- | --- | --- | --- | --- |
|  | **n (% per column)** | **Median G6PD activity in U/gHb (IQR, min - max)** | **n (% per column)** | **Median G6PD activity in U/gHb (IQR, min-max)** | ***p*** |
| **Mahidol homozygous** | 11 (68.8) | 0.96 (0.46 – 1.68, 0.25 – 1.95) | 17 (73.9) | 0.88 (0.67 – 1.04, 0.54 – 1.72) | 0.725 |
| **Mahidol heterozygous** | 5 (31.3) | 4.20 (3.95 – 5.35, 3.85 – 6.03) | 2 (8.7) | 6.61 (6.51 – 6.70, 6.51 – 6.70) | 0.071 |
| **Mediterranean heterozygous** | 0 (0.0) | n=0 | 1 (4.4) | 2.34 (n=1) | 0.398 |
| **Orissa heterozygous** | 0 (0.0) | n=0 | 3 (13.0) | 1.54 (1.33 – 7.44, 1.33 – 7.44) | 0.133 |
| **Total** | 16 (100.0) | - | 23 (100.0) | - | - |

**References**

1.	Battle KE, Lucas TCD, Nguyen M, Howes RE, Nandi AK, Twohig KA, et al. Mapping the global endemicity and clinical burden of Plasmodium vivax, 2000-17: a spatial and temporal modeling study. Lancet. 2019.

2.	Price RN, Commons RJ, Battle KE, Thriemer K, Mendis K. Plasmodium vivax in the Era of the Shrinking P. falciparum Map. Trends in parasitology. 2020;36(6):560-70.

3.	Dini S, Douglas NM, Poespoprodjo JR, Kenangalem E, Sugiarto P, Plumb ID, et al. The risk of morbidity and mortality following recurrent malaria in Papua, Indonesia: a retrospective cohort study. BMC Med. 2020;18(1):28.

4.	Phru CS, Kibria MG, Thriemer K, Chowdhury MU, Jahan N, Aktaruzzaman MM, et al. Case Report: A Case of Primaquine-Induced Hemoglobinuria in Glucose-6-Phosphate Dehydrogenase-Deficient Malaria Patient in Southeastern Bangladesh. The American journal of tropical medicine and hygiene. 2019.

5.	WHO. Guide to G6PD deficiency rapid diagnostic testing to support P. vivax radical cure. In: Organization WH, editor. Geneva, switzerland2018.

6.	Cappellini MD, Fiorelli G. Glucose-6-phosphate dehydrogenase deficiency. Lancet. 2008;371(9606):64-74.

7.	Howes RE, Battle KE, Satyagraha AW, Baird JK, Hay SI. G6PD deficiency: global distribution, genetic variants and primaquine therapy. Adv Parasitol. 2013;81:133-201.

8.	Mbanefo EC, Ahmed AM, Titouna A, Elmaraezy A, Trang NT, Phuoc Long N, et al. Association of glucose-6-phosphate dehydrogenase deficiency and malaria: a systematic review and meta-analysis. Sci Rep. 2017;7:45963.

9.	Robinson KM, Yang W, Haidar CE, Hankins JS, Jay DW, Kornegay N, et al. Concordance between glucose-6-phosphate dehydrogenase (G6PD) genotype and phenotype and rasburicase use in patients with hematologic malignancies. Pharmacogenomics J. 2019;19(3):305-14.

10.	Roh ME, Oyet C, Orikiriza P, Wade M, Mwanga-Amumpaire J, Boum Y, 2nd, et al. Screening for Glucose-6-Phosphate Dehydrogenase Deficiency Using Three Detection Methods: A Cross-Sectional Survey in Southwestern Uganda. The American journal of tropical medicine and hygiene. 2016;95(5):1094-9.

11.	Shah SS, Macharia A, Makale J, Uyoga S, Kivinen K, Craik R, et al. Genetic determinants of glucose-6-phosphate dehydrogenase activity in Kenya. BMC Med Genet. 2014;15:93.

12.	Johnson MK, Clark TD, Njama-Meya D, Rosenthal PJ, Parikh S. Impact of the method of G6PD deficiency assessment on genetic association studies of malaria susceptibility. PloS one. 2009;4(9):e7246.

13.	Chu CS, Bancone G, Nosten F, White NJ, Luzzatto L. Primaquine-induced haemolysis in females heterozygous for G6PD deficiency. Malaria journal. 2018;17(1):101.

14.	Luzzatto L, Arese P. Favism and Glucose-6-Phosphate Dehydrogenase Deficiency. The New England journal of medicine. 2018;378(1):60-71.

15.	White NJ. Malaria parasite clearance. Malaria journal. 2017;16(1):88.

16.	Anstey NM, Russell B, Yeo TW, Price RN. The pathophysiology of vivax malaria. Trends in parasitology. 2009;25(5):220-7.

17.	Douglas NM, Anstey NM, Buffet PA, Poespoprodjo JR, Yeo TW, White NJ, et al. The anaemia of Plasmodium vivax malaria. Malaria journal. 2012;11:135.

18.	Ley B, Alam MS, Thriemer K, Hossain MS, Kibria MG, Auburn S, et al. G6PD Deficiency and Antimalarial Efficacy for Uncomplicated Malaria in Bangladesh: A Prospective Observational Study. PloS one. 2016;11(4):e0154015.

19.	Ley B, Alam MS, O'Donnell JJ, Hossain MS, Kibria MG, Jahan N, et al. A Comparison of Three Quantitative Methods to Estimate G6PD Activity in the Chittagong Hill Tracts, Bangladesh. PloS one. 2017;12(1):e0169930.

20.	Heather Scobie SA, Malathi Ram, Md Sharif Hossain, Chai Shwai Prue, Jacob Khyang, David Sullivan, Peter Kim Streatfield, Wasif Ali Khan, David Sack Initial Demographic Surveillance System Report for the icddr,b Bandarban Field Site. Bangladesh: International Center For Diarrheal Disease Research, Bangladesh; 2012 March 2012. Contract No.: 119.

21.	Khan WA, Sack DA, Ahmed S, Prue CS, Alam MS, Haque R, et al. Mapping hypoendemic, seasonal malaria in rural Bandarban, Bangladesh: a prospective surveillance. Malaria journal. 2011;10:124.

22.	Streatfield PK, Khan WA, Bhuiya A, Alam N, Sie A, Soura AB, et al. Cause-specific mortality in Africa and Asia: evidence from INDEPTH health and demographic surveillance system sites. Glob Health Action. 2014;7:25362.

23.	Galagan SR, Prue CS, Khyang J, Khan WA, Ahmed S, Ram M, et al. The practice of jhum cultivation and its relationship to Plasmodium falciparum infection in the Chittagong Hill Districts of Bangladesh. The American journal of tropical medicine and hygiene. 2014;91(2):374-83.

24.	Alam MS, Kibria MG, Jahan N, Thriemer K, Hossain MS, Douglas NM, et al. Field evaluation of quantitative point of care diagnostics to measure glucose-6-phosphate dehydrogenase activity. PloS one. 2018;13(11):e0206331.

25.	Domingo GJ, Satyagraha AW, Anvikar A, Baird K, Bancone G, Bansil P, et al. G6PD testing in support of treatment and elimination of malaria: recommendations for evaluation of G6PD tests. Malaria journal. 2013;12:391.

26.	Kashmoola MA, Eissa AA, Al-Takay DT, Al-Allawi NA. Molecular Characterization of G6PD Deficient Variants in Nineveh Province, Northwestern Iraq. Indian J Hematol Blood Transfus. 2015;31(1):133-6.

27.	Sarker SK, Islam MT, Eckhoff G, Hossain MA, Qadri SK, Muraduzzaman AK, et al. Molecular Analysis of Glucose-6-Phosphate Dehydrogenase Gene Mutations in Bangladeshi Individuals. PloS one. 2016;11(11):e0166977.

28.	Ley B, Rijal KR, Marfurt J, Adhikari NR, Banjara MR, Shrestha UT, et al. Analysis of erroneous data entries in paper based and electronic data collection. BMC Res Notes. 2019;12(1):537.

29.	von Seidlein L, Auburn S, Espino F, Shanks D, Cheng Q, McCarthy J, et al. Review of key knowledge gaps in glucose-6-phosphate dehydrogenase deficiency detection with regard to the safe clinical deployment of 8-aminoquinoline treatment regimens: a workshop report. Malaria journal. 2013;12:112.

30.	Luzzatto L, Nannelli C, Notaro R. Glucose-6-Phosphate Dehydrogenase Deficiency. Hematol Oncol Clin North Am. 2016;30(2):373-93.

31.	Minucci A, Giardina B, Zuppi C, Capoluongo E. Glucose-6-phosphate dehydrogenase laboratory assay: How, when, and why? IUBMB Life. 2009;61(1):27-34.

32.	Watson J, Taylor WR, Menard D, Kheng S, White NJ. Modelling primaquine-induced haemolysis in G6PD deficiency. Elife. 2017;6.

33.	Haldar K, Mohandas N. Malaria, erythrocytic infection, and anemia. Hematology Am Soc Hematol Educ Program. 2009:87-93.

34.	Lamikanra AA, Brown D, Potocnik A, Casals-Pascual C, Langhorne J, Roberts DJ. Malarial anemia: of mice and men. Blood. 2007;110(1):18-28.

35.	Hillman RS. Characteristics of marrow production and reticulocyte maturation in normal man in response to anemia. J Clin Invest. 1969;48(3):443-53.

36.	Beutler E, Dern RJ, Alving AS. The hemolytic effect of primaquine. IV. The relationship of cell age to hemolysis. J Lab Clin Med. 1954;44(3):439-42.

37.	WHO. Guidelines for the Treatment of Malaria. 3rd edition Geneva: World Health Organization (WHO); 2015 [3rd edition:[Available from: <http://apps.who.int/medicinedocs/documents/s21839en/s21839en.pdf>.

38.	WHO. Guidelines for the treatment of malaria - 3rd Edition. Switzerland: WHO; 2015.

39.	Alving AS, Johnson CF, Tarlov AR, Brewer GJ, Kellermeyer RW, Carson PE. Mitigation of the haemolytic effect of primaquine and enhancement of its action against exoerythrocytic forms of the Chesson strain of Piasmodium vivax by intermittent regimens of drug administration: a preliminary report. Bull World Health Organ. 1960;22:621-31.

40.	Dern RJ, Beutler E, Alving AS. The hemolytic effect of primaquine. II. The natural course of the hemolytic anemia and the mechanism of its self-limited character. J Lab Clin Med. 1954;44(2):171-6.

41.	Ashley EA, Recht J, White NJ. Primaquine: the risks and the benefits. Malaria journal. 2014;13:418.

42.	Ley B, Winasti Satyagraha A, Rahmat H, von Fricken ME, Douglas NM, Pfeffer DA, et al. Performance of the Access Bio/CareStart rapid diagnostic test for the detection of glucose-6-phosphate dehydrogenase deficiency: A systematic review and meta-analysis. PLoS medicine. 2019;16(12):e1002992.

43.	Chu CS, Bancone G, Moore KA, Win HH, Thitipanawan N, Po C, et al. Haemolysis in G6PD Heterozygous Females Treated with Primaquine for Plasmodium vivax Malaria: A Nested Cohort in a Trial of Radical Curative Regimens. PLoS medicine. 2017;14(2):e1002224.

44.	Gunasekera W, Premaratne RG, Weerasena O, Premawansa WS, Handunnetti SM, Fernando SD. Utility of pf/pan RDT for diagnosis in the prevention of re-establishment of malaria in Sri Lanka. Pathog Glob Health. 2018;112(7):360-7.

45.	Ruwende C, Khoo SC, Snow RW, Yates SN, Kwiatkowski D, Gupta S, et al. Natural selection of hemi- and heterozygotes for G6PD deficiency in Africa by resistance to severe malaria. Nature. 1995;376(6537):246-9.

46.	Guindo A, Fairhurst RM, Doumbo OK, Wellems TE, Diallo DA. X-linked G6PD deficiency protects hemizygous males but not heterozygous females against severe malaria. PLoS medicine. 2007;4(3):e66.

47.	Clarke GM, Rockett K, Kivinen K, Hubbart C, Jeffreys AE, Rowlands K, et al. Characterisation of the opposing effects of G6PD deficiency on cerebral malaria and severe malarial anaemia. Elife. 2017;6.

48.	Howes RE, Dewi M, Piel FB, Monteiro WM, Battle KE, Messina JP, et al. Spatial distribution of G6PD deficiency variants across malaria-endemic regions. Malaria journal. 2013;12:418.

49.	Plewes K, Soontarawirat I, Ghose A, Bancone G, Kingston HW, Herdman MT, et al. Genotypic and phenotypic characterization of G6PD deficiency in Bengali adults with severe and uncomplicated malaria. Malaria journal. 2017;16(1):134.
