# Supplementary material for: Glucose-6-phosphate dehydrogenase activity in individuals with and without malaria: Analysis of clinical trial, cross-sectional and case–control data from Bangladesh
Source: PLoS Med. 2021 Apr 23;18(4):e1003576. doi: 10.1371/journal.pmed.1003576 (PMC8064587; doi:10.1371/journal.pmed.1003576)
Supplement: Attachment — Submitted filename: Rebuttal_Ley_v0.2.docx [file pmed.1003576.s011.docx]

**Requests from the editors:**

***PLOS Medicine style for the title is normally to include some designation of the study design for the methodological approach (usually after a colon following the study question/objective) - here I appreciate that is tricky because three groups are involved (trial, x-sectional and case-control). If the authors are amenable however we'd suggest stating that in the title subtitle (eg, "Variability of Glucose-6-Phosphate Dehydrogenase activity in individuals with and without malaria: analysis of trial, cross-sectional and case-control data" for example.**

We have changed the title, which now reads: “*Glucose-6-Phosphate Dehydrogenase activity in individuals with and without malaria in Bangladesh: analysis of clinical trial, cross-sectional and case-control data*”

***There is some confusion in the abstract where initially the authors state they have analysed patient groups per the above frameworks (trial, cross-sectional and case-control data) but later on the authors suggest they are analysing a prospective cohort. For consistency the authors need to be clear, and also to help the readers follow which of the groups they are referring to.**

We have revised the text which now reads: “*In the case control study, the median G6PD activity was 10.3 U/gH (IQR: 8.8 to 12.2) in the 253 patients with a history of malaria and 10.2 U/gHb (IQR: 8.7 to 11.8) in the 253 individuals without a history of malaria (p=0.323).*“ (lines 46 – 48)

**The Author Summary section would need some stylistic changes to fit our usual format, please check the guidelines here:**

We have changed the authors summary to match the stylistic requirements.

***Where the authors mention the Ley et al accepted paper in PLOS NTD's, we'd suggest instead noting the accepted paper in the reference list as for the other papers - give the author list, paper title, and then PLOS NTD's "accepted for publication". PLOS Med reference policy allows for inclusion in the reference list of accepted publications**

The respective paper is published by now and has been included as a reference 20.

***Although this is complex because the paper uses data from three separate participant groups, we'd suggest aiming to ensure the study is reported according to the STROBE guideline, and include the completed STROBE checklist as Supporting Information. The important thing here would be to ensure that the elements of the guideline are covered in the reporting in the paper, rather than to try to fill out the checklist in a way that reflects all three study populations. We'd also suggest including this statement (or something along these lines) in the methods section of the paper - "This study is reported as per the Strengthening the Reporting of Observational Studies in Epidemiology (STROBE) guideline (S1 Checklist)."**

We have attached the completed STROBE list to the re-submission as S1 File.

**Reviewer 1 (statistical review):**

**Page 9 I can see that categorising G6PD activity may be helpful for presentation but it does miss the chance to see whether these cut-offs are in fact appropriate. Categorising an essentially continuous variable wastes information (Altman and Royston, 2006; Royston et al., 2006) and leads to models which are often implausible as they predict the effect remaining at within categories and then jumping to a new value at the category boundary. The position would of course have been worse if the choice had been made with knowledge of the data (Altman et al., 1994).**

We agree that categorizing continuous data results in loss of information, however these categories are of practical relevance since the corresponding cut-offs (<30% activity and <70% activity) are specifically used to guide primaquine and tafenoquine treatment. Current guidelines recommend for patients with ≤30% activity weekly primaquine treatment for 8 weeks, for patients with >30% a standard course of primaquine treatment (7- 14 days), while patients with >70% are eligible for single dose tafenoquine treatment.

We have rephrased the respective section, which now reads:

*“Current treatment guidelines recommend that individuals with G6PD activities below 30% are categorized as G6PD deficient, those with activities between 30% and below 70% activity as G6PD intermediate, and those with activities at or above 70% as G6PD normal (5, 32, 33)”* (lines 212 – 215)

We have also added two references for the WHO guidelines (reference #5 and #33)

**Page 10 Strictly speaking multivariable is meant here and elsewhere (Hidalgo and Goodman, 2013). Multivariate means multiple variables on the left-hand side.**

We have changed this to “multivariable” throughout the manuscript (lines 224, 318, 354, 424).

**Page 10 I do not see a clear scientific or clinical justification put forward for doing variable selection. Selecting a subset of variables in a way driven by the data leads to a model which is unlikely to replicate (Babyak, 2004). It would be better to fit a model which relied on theory or, if the goal is just adjustment, to fit a model with all the predictors.**

We have revised the manuscript accordingly. The methods now read: “*For each spectrophotometry assay, a multivariable regression model was developed to predict non-normalized G6PD activity (in U/dL), including Hb, sex, and G6PD genotype as biologically plausible independent variables. In addition malaria status (defined as negative, only positive by PCR or positive by microscopy or RDT) was included in the model comparing participants with and without parasitemia, while history of malaria was included as a predictor in the model corresponding to the case control study*. *“ (*Lines 223 – 229).

The overall outcome and conclusions of each model remain unchanged. We have updated the results section accordingly (lines 316 – 320, table 5 and lines 353 – 357, table 7).

**Page 11 For completeness I think the y-axis should be labelled.**

The labelling of the y-axis was lost when converting the manuscript to pdf. The y-axis of figure 1 is now clearly labelled as “Percent (%)”.

**Page 12, Table 1 I wonder whether it might be better to show the difference in means along with its confidence interval rather than confidence intervals for the individual means?**

We have added the suggested column in table 1 and table 6.

**Page 15, Figure 2 We definitely do need a label for the y-axis here. The reader is going to make the assumption that all four plots are on the same scale but if these are raw frequencies then with sample sizes varying from 48 to 595 that is clearly not the case.**

We think the reviewer refers to figure 3. The y-axis labelling was lost when the manuscript was converted to a pdf, the y-axis reflects % of each cohort

**Page 16 What was the value of the correlation? A confidence interval might also be helpful to get some idea of precision so we can evaluate the import of its failure to meet some arbitrary level of statistical significance.**

We have added the correlation coefficient as requested . The sentence now reads: “*There was no significant correlation between peripheral parasitemia and G6PD activity (r_s_=0.001, p=0.988), and this remained after stratifying by species (all p>0.05).*” (lines 302 – 304)

**Page 17 With only 2 or 3 observations why not just give them? The IQR and range use more space to give us the same information.**

Although the number of participants with confirmed genotype and parasitemia are low, the number of aparasitemic participants with an identified variant is much larger. Hence we would prefer to keep the format of table 4 as presented.

**Page 18 Perhaps give the unit for the continuous ones rather than just saying continuous so we can see what the estimated coeficient refers to? I am not sure why weight had to be categorised into two categories when it is inherently continuous. In general for categorical variables we could use the explicit reference category being quoted too.**

We have added units for Hb in Table 5 and removed the weight variable (see point on selection of variables for the regression model).

**Page 19, Figure 4 Label for y-axis again.**

The y-axis labelling was lost when the manuscript was converted to a pdf, the y-axis reflects % of each cohort

**Page 20 As for page 18. This does raise the issue of why the variants displayed here are not the same ones as on page 18. Are these the totality of possible variants in that sample or are the omitted ones an artefact of using variable selection? If the latter that seems to me a further argument against variable selection.**

Kindly see the point above on variable selection. Following the reviewers suggestion we have now applied the same predictors to both models.

**Point of more substance**

**I assume from the use of quantiles on page 11 that a non-parametric test was used here although not the Wilcoxon signed - rank test specified in the methods since these are independent samples. It would really be helpful here to have more on the differences especially given the authors comments on page 22. Then we could see whether there is a very precise estimate of the differences. Even without that information I suspect the authors are correct that any differences are in fact not of major scientific importance. Why not use the Hodges-Lehmann estimator which does yield a confidence interval? There are other options of course.**

We would like to thank the reviewer for the advice. We used the Kruskal -Wallis equality of populations rank test but have now applied the method suggested by the reviewer. The difference between control readings was not significant when stratifying for control type. These changes are reflected in the methods (lines 220 - 222), the results section (lines 245 – 247) and the limitations section (lines 396 – 398).

**Summary**

**Mostly for clarification. Relying so much on p-values rather than measures of effect size could, and I believe should, be avoided.**

As suggested, we have added absolute differences and corresponding 95%CI in addition to the p-values.

**Reviewer #2:**

**G6PD activity is dependent on genotype and RBC's age. The authors tried to clarify the real time enzymatic activity in the individuals who are exposed to acute malaria using a DSS cohort and a clinical efficacy trial in the endemic area of Bangladesh. The authors found the G6PD activity showed a significant increase in malaria positive patients compared with malaria negative or asymptomatic PCR positives.**

**Combined with a case control study of past malaria history positives, the authors concluded that the G6PD activity increased after the hemolysis of parasitized old RBCs which is independent from genotype origin. Field study and related laboratory protocol including enzymatic estimation and genotyping are reasonable. However, the conclusion could not be completely supported by their findings. To make more convincing discussion and recommendation, several major points should be clarified.**

**Major comment**

**1.	Case-control study needs more details because this is essential to lead to their conclusion that malaria infection increased the enzyme activity, For the reviewer, there are a lot of questions about the criteria of malaria history positive or negative.**

We have revised the background section which now reads: “*In 2009, a prospective demographic surveillance system (DSS) was established in the Bandarban district amongst a population of 20,558 and is currently part of the INDPETH network (22-24). Coordinates of every household within the surveillance area were recorded and all households were visited between 2009 and 2010 to enroll participants (25). All participants were assigned a unique identifier (ID) at the initial visit and then reviewed every three months, until December 2014, then every four months in 2015, and bi-annually since 2016 . At the end of 2018, a total of 19,117 people were under surveillance. At each follow up visit a history of malaria was recorded, along with in- and outmigration, births, deaths, and changes of address. Febrile participants were asked to notify the study clinical staff, who then provided a malaria rapid diagnostic test (RDT, FalciVax, Zephyr Biomedicals, Goa, India). RDT positive participants were prescribed free antimalarial treatment according to national treatment guidelines, the episode of malaria was recorded, and the participant was reviewed at the end of treatment to ensure clinical recovery.*

*This cohort was used to generate a case-control study comprising of individuals, who were all afebrile and aparasitemic at the time of enrolment.” (lines 140 – 153)*

We also added the following section in the discussion: “*A subset of 100 participants from each case and control arm were included in a separate study of the perceptions of malaria (48). A total of 6 participants from the control arm recalled having had malaria previously, whereas only one case could not recall any previous episodes of malaria. While these findings are vulnerable to recall and response bias, they suggest that the majority of participants were categorized correctly.”* (lines 404 – 409)

Added Reference (48):

*Matin MA, Sarkar NDP, Phru CS, Ley B, Thriemer K, Price RN, et al. Precarity at the Margins of Malaria Control in the Chittagong Hill Tracts in Bangladesh: A Mixed-Methods Study. Pathogens. 2020;9(10).*

**Even the protective effect of G6PDd is controversial in Asia and Africa especially against different species such as P. falciparum and P. vivax. Please describe how many Pf and Pv. The endemicity of malaria is not clear there. Please describe how many positive cases observed in the cohort population.**

We have added this information for the efficacy trial and cross sectional survey:

“*Of the 182 participants with patent parasitemia 117 (64.3%) were infected with P. falciparum, 54 (29.7%) with P. vivax and 11 (6.0%) with a mixed P. falciparum and P.vivax infection. Among the 36 participants with sub-patent parasitemia 17 (47.2%) had a P. falciparum infection, 16 (44.4%) a P. vivax infection, and 3 (8.3%) were positive for both species.”* (lines 253 – 257)

In the case control study all participants were free of malaria as per inclusion criteria.

**Also the authors should explain the seasonality and annual changes of the endemicity.**

We have added this information and a corresponding reference: “*Malaria in Bangladesh is highly seasonal, with more than 80% of all cases reported during the rainy season from May to October, 80% to 90% of all reported cases are attributed to P. falciparum, the majority of other cases is due to P. vivax (18)*.” (lines: 111-113)

Reference:

18.	Noe A, Zaman SI, Rahman M, Saha AK, Aktaruzzaman MM, Maude RJ. Mapping the stability of malaria hotspots in Bangladesh from 2013 to 2016. Malaria journal. 2018;17(1):259.

2**.	The comparison between G6PD low, intermediate and normal groups for their malaria infection strongly suggested a protective effect of low enzymatic activity which is opposite to the description at page 21, line 356. For the reviewer, the protective effect looks very clear. However, the authors denied this possibility. Please explain the reason why without mentioning the case control study.**

We agree that G6PD deficiency may have a protective effect against malaria and this would be explain its predominance in malaria endemic areas. However we undertook the case control study to demonstrate the degree of this association and whether it could account for difference in G6PD prevalence in patients with and without malaria.

Our results highlight that patients with patent malaria have significantly higher G6PD activity and this is unlikely to be attributable to the modest degree of protection that maybe afforded by being G6PD deficient.

“*There was no significant difference between cases and controls in the proportion of patients categorized as G6PD normal, intermediate, or deficient (p=0.328), although cases were at lower risk than controls of having a G6PD deficiency defined at the 30% threshold (OR=0.60, 95%CI: 0.27 to 1.29) and at the 70% threshold (OR=0.71, 95%CI: 0.41 to 1.23) this did not reach statistical significance (p=0.155 and p=0.241 respectively);* *…”* (lines 339 to 343).

We do not rule out a protective effect in lines 410-418: “*There was a trend for individuals without a history of malaria to be G6PD intermediate or severely deficient, as has been observed by others (8, 49-51). A recent publication among more than 17,000 infants and neonates in Kenya found the odds in heterozygous females of being admitted to hospital with a malaria positive microscopy slide to be slightly lower (OR = 0.79, 95%CI: 0.68 to 0.93, p=0.004) compared to G6PD normal individuals, in our analysis the protective effect was modest (odds ratio less than 0.7) and did not reach statistical significance (52). If a protective effect of G6PDd against malaria was present it is likely to be a minor contributor to G6PD status and would not account for asymptomatic and parasite-free individuals being 4 to 9-fold more likely to be diagnosed as G6PD deficient in the clinical trial and cross-sectional survey.”*

**3.	Regarding genetic polymorphism, the Mahidol variant looked a major one. Please describe more about the genotype information about female population in the Figure 3 separating by 30,70,100% activity.**

Information on phenotypic G6PD activity of heterozygous females carrying the Mahidol variant is included in table 4 and S2 Table and S3 Table.

**Minor comment**

1.	**In the title, is it necessary to use Variability?**

We have changed the title, which now reads: “*Glucose-6-Phosphate Dehydrogenase activity in individuals with and without malaria in Bangladesh: analysis of clinical trial, cross-sectional and case-control data*”

2.	**Please explain the relationship between acute malaria infection time course and PQ treatment.**

G6PD activity was measured before treatment with PQ. In the efficacy trial, it was measured on the day of presentation, but radical cure was only started on day 2. In the cross-sectional survey parasitemic participants provided a sample for G6PD measurement before referral to the closest medical facility.

We have clarified this for the efficacy study: “*Venous blood samples were collected for phenotypic and genotypic G6PD testing on day of enrolment,* *prior to administration of primaquine which was commenced 48 hours after enrolment*.” (lines 125 – 127)

…and for the cross-sectional survey: “*Participants with microscopic parasitemia provided a sample for laboratory analysis prior to being referred to the closest treatment facility where they were treated according to national treatment guidelines.*” (lines 135 – 137)

**3.	Please cite any reference to give an evidence of increased G6PD activity in experimental malaria model or in human study. Or is it possible to show any information about the enzymatic activity of the same individual when infected or not?**

We are not aware of any respective data that has been published to date but agree that a longitudinal cohort study is the next step to confirm these findings. We highlight this in the conclusion where it says:

*“If confirmed prospectively, 8-aminoquinoline based radical cure may be safer than assumed in patients with clinical malaria, compared to individuals who have submicroscopic parasitaemia or who are aparasitaemic..”* (line 427 - 429)

4**.	Page 21, line 378. I need a detailed description about this odds. It is very difficult for me to understand.**

This statement discusses the results from the efficacy trial and the cross sectional survey in which females (who are most likely to have intermediate deficiency) had the greatest shift in their G6PD activity when they have malaria.

We have rephrased the sentence as follows:

*“Our analysis highlights that compared to sub-patent or aparasitemic females, females with microscopic parasitaemia were 14 times less likely to be intermediate or severely deficient; the corresponding ratio for males was 3.6. We hypothesize that this is due to hemolysis of G6PD deficient erythrocytes in mosaic heterozygous females with detectable parasitaemia. If confirmed, our findings suggest that the risk of PQ induced haemolysis in heterozygous females with malaria would be considerably less than that predicted by cross sectional surveys of healthy populations.”* (lines 387 – 393)

**Reviewer #3:**

**Accept with suggestions for a few minor revisions**

**The paper by Benedikt Ley et al. of the Variability of Glucose-6-Phosphate Dehydrogenase activity in individuals with and without malaria in the Chittagong Hill Tracts of Bangladesh:**

**The authors measured G6PD activities among**

**-	individuals with and without malaria and found G6PD activities to be significantly higher among malaria patients.**

**-	individuals enrolled healthy with and without a history of malaria to assess whether people with low G6PD activities were less likely to contract malaria. They found thatG6PD activities did not differ significantly.**

**They conclude that G6PD activity increases during a malaria infection and the observed clinically relevant difference is not due to people with lower G6PD activities being less likely to contract malaria.**

**The title and abstract are appropriate for the content of the text. Furthermore, the article is well constructed and analysis was well performed.**

**The authors demonstrated that their study highlights the potential impact of acute malaria on G6PD status, which may influence treatment allocation of an 8-aminoquinoline drug. this a good alternative because the cure may be safer than assumed in patients with clinical malaria, compared**

**The article highlights important data to know, however, we have some suggestions.**

**1.	The document: need a few minor amendment (formatting, Typos, spelling, grammar, and phrasing issues). I have propose some suggestions.**

Many thanks for the thorough review, we have revised the manuscript to address the proposed changes.

**2.	The authors should clarify and give more explanation in the discussion sections: paragraph bellow:**

**"During laboratory quality control testing, there was a significant difference in the enzyme activity of controls between the efficacy study and the cross-sectional survey that may have influenced the direct comparison of results**

We have already addressed this in the response to the statistical Reviewer 1 who suggested a more appropriate statistical test. The results don’t differ significantly.

**3.	The authors should confirm if their findings indicate a substantial variation in G6PD measurements between the different methods used and different studies. We will suggest that the authors give more explication because caution is advised in comparing findings based on absolute G6PD activity measurements across studies.**

We agree with the reviewer that assay methodology is an important confounder. Hence we have not compared studies that used different spectrophotometry assays. Although the efficacy trial and the cross-sectional survey were not done at the same time, spectrophotometry control readings were run in parallel to the experimental measurements, and demonstrated that procedures did not vary, the results are therefore comparable. We have clarified this by adding the following sentence (including Pfeffer et al as reference as suggested below):

*“Since absolute values between different spectrophotometry assays can vary, results from the Randox and Pointe Scientific assays were not compared directly but rather used to categorize participants according to the percentage of normal enzyme activity (27).”* (lines 184 – 187)

**4.	The authors should give if possible the mean temperature and mean precipitation during the different periods of three studies (The main climatic factors that influence the transmission of malaria are temperatures and precipitation)**

Whilst temperature variation may fluctuate with season and can influence G6PD measurements we believe it is less relevant to the current analysis. Spectrophotometry was undertaken in an air conditioned laboratory and the consistent measurements of the control isolates highlight the stability of our results. Precise data on the background climatic variation is not available and it is not apparent why this would influence our results.

**5.	The authors have identified much literature to relates to this research. However, we suggest that the authors using also the below some publications to explain the introduction**

**1)	Pfeffer DA, Ley B, Howes RE, Adu P, Alam MS, Bansil P, et al. (2020) Quantification of glucose-6-phosphate dehydrogenase activity by spectrophotometry: A systematic review and metaanalysis. PLoS Med 17(5): e1003084.** [**https://doi**](https://doi)**. org/10.1371/journal.pmed.1003084**

Added as reference 27

**2)	Kießling et al. Malar J (2018) 17:358** [**https://aus01.safelinks.protection.outlook.com/?url=https%3A%2F%2Fdoi.org%2F10.1186%2Fs12936-018-2510-3&amp;data=04%7C01%7Cbenedikt.ley%40menzies.edu.au%7C188947e062c74ac4b6cf08d89de5a7bd%7C9f2487678e1a42f3836fc092ab95ff70%7C0%7C0%7C637432957165395157%7CUnknown%7CTWFpbGZsb3d8eyJWIjoiMC4wLjAwMDAiLCJQIjoiV2luMzIiLCJBTiI6Ik1haWwiLCJXVCI6Mn0%3D%7C1000&amp;sdata=GleSzsORvWDKjzTGB7MpG5A2%2FXI5jR0EfirYH0ZFyEs%3D&amp;reserved=0**](https://aus01.safelinks.protection.outlook.com/?url=https%3A%2F%2Fdoi.org%2F10.1186%2Fs12936-018-2510-3&amp;data=04%7C01%7Cbenedikt.ley%40menzies.edu.au%7C188947e062c74ac4b6cf08d89de5a7bd%7C9f2487678e1a42f3836fc092ab95ff70%7C0%7C0%7C637432957165395157%7CUnknown%7CTWFpbGZsb3d8eyJWIjoiMC4wLjAwMDAiLCJQIjoiV2luMzIiLCJBTiI6Ik1haWwiLCJXVCI6Mn0%3D%7C1000&amp;sdata=GleSzsORvWDKjzTGB7MpG5A2%2FXI5jR0EfirYH0ZFyEs%3D&amp;reserved=0)

This article describes a G6PD survey in healthy volunteers from Ethiopia, and thus is less relevant to our analysis. Kindly also see our reply below.

**3)	Emilie S. Badoum, Samuel S. Sermé, Jean B. Yaro, Sam. A Coulibaly, Désiré Kargougou, Amidou Diarra, Amidou Z Ouédraogo, Lankoande Malik, Issa Nébié, Issiaka Soulama, Alphonse Ouédraogo, Alfred B. Tiono, Yves Traoré, Sodiomon B. Sirima, Edith C. Bougouma* Abnormalities of hemoglobin and Glucose-6-Phosphate-Dehydrogenase deficiency in children with uncomplicated malaria and living in Banfora and Saponé, two different malaria setting of Burkina Faso International Journal of TROPICAL DISEASE & Health 37(3): 1-10, 2019; Article no.IJTDH.50379 ISSN: 2278-1005, NLM ID: 101632866**

This study describes the prevalence of G6PD variants in children with *P. falciparum* infection in Burkina Faso.

We don’t think its appropriate to compare prevalence of G6PD deficiency between Bangladesh, Ethiopia and Burkina Faso considering the different background prevalence of G6PD deficiency in both countries.
