# Supplementary material for: Glucose-6-phosphate dehydrogenase activity in individuals with and without malaria: Analysis of clinical trial, cross-sectional and case–control data from Bangladesh
Source: PLoS Med. 2021 Apr 23;18(4):e1003576. doi: 10.1371/journal.pmed.1003576 (PMC8064587; doi:10.1371/journal.pmed.1003576)
Supplement: Attachment — Submitted filename: Rebuttal_Ley_v0.3.docx [file pmed.1003576.s012.docx]

**Requests from the editors:**

*Many thanks for the suggested changes, we have addressed these below. We have also added a recent publication on the protective effect of g6PD deficiency against P. vivax (reference 52) in the discussion (lines 417 to 426), where it now reads: “A recent publication among more than 17,000 infants and neonates in Kenya found the odds in heterozygous females of being admitted to hospital with a malaria positive microscopy slide to be lower (OR = 0.79, 95%CI: 0.68 to 0.93, p=0.004) compared to G6PD normal individuals, a recent article from Afghanistan reported an even greater protective effect against P. vivax malaria for the Mediterranean variant (76% in hemizygous males and 55% in females) [52,53].”. (underlined parts are new additions)*

*Reference:*

*52.	Awab GR, Aaram F, Jamornthanyawat N, Suwannasin K, Pagornrat W, Watson JA, et al. Protective effect of Mediterranean-type glucose-6-phosphate dehydrogenase deficiency against Plasmodium vivax malaria. Elife. 2021;10.*

•	**Was the clinical trial registered in a recognized database?**

*The trial was registered with clinicaltrials.gov as NCT02389374. We have added this information under the trail description in lines 123 – 125: “The prospective, single arm, clinical trial was conducted between September 2014 and February 2015 in Alikadam in the district of Bandarban (CHT) and was registered with the clinical trials website (http://www.clinicaltrials.gov/ct) as NCT02389374.“*

•	**At key points in the paper, i.e., in the abstract, methods section and discussion, please adapt the description "clinical trial" to provide slightly more information on the study design, e.g., "prospective, single-arm clinical trial"**

*We have added the terms” prospective, single arm” in the abstract (line 33), methods (line 123), and in the discussion (line 401)*

•	**Please reduce the length of your abstract, if possible.**

*We have shortened the abstract to 406 words.*

•	**Please quote study dates in your abstract; along with number of participants in the individual studies and aggregate demographic details**

*We have added dates and sample size per study in the abstract.*

**•	At line 98, it may be that "lyonization" should take an initial capital, or the alternative term used.**

*We have changed this to an initial capital (line 96)*

**•	Please remove trademarks from your manuscript, e.g. at line 159**

*We have removed the trademark symbol in line 162 and have checked the rest of the manuscript accordingly.*

**•	Please remove information on funding from the title page and end of the main text. This information will appear in the article metadata upon publication, via information in the submission form**

*Removed accordingly*

•	**Please hyphenate "case-control" consistently.**

*Hyphenated throughout the manuscript.*

•	**Please add the following statement, or similar, to the Methods: "This study is reported as per the Strengthening the Reporting of Observational Studies in Epidemiology (STROBE) guideline (S1 Checklist)."**

*We have added the suggested sentence to the methods section (line 118 – 120)*

•	**Please indicate in the figure caption the meaning of the red vertical lines in Fig. 3 and Fig. 4**

*Added in lines 286 and 349.*

•	**Please define the abbreviations in all tables and figures, including: G6PD, RDT, PCR, IQR**

*Added for all tables and figures*

•	**Please provide titles and legends for all figures (including those in Supporting Information files).**

*We double checked, all figures and tables have a title and legend*

•	**Please use the "Vancouver" style for reference formatting and see our website for other reference guidelines** https://aus01.safelinks.protection.outlook.com/?url=https%3A%2F%2Fjournals.plos.org%2Fplosmedicine%2Fs%2Fsubmission-guidelines%23loc-references&amp;data=04%7C01%7Cbenedikt.ley%40menzies.edu.au%7Cd44162c7bc764f3d5d1a08d8d1bb528c%7C9f2487678e1a42f3836fc092ab95ff70%7C0%7C0%7C637489949782771837%7CUnknown%7CTWFpbGZsb3d8eyJWIjoiMC4wLjAwMDAiLCJQIjoiV2luMzIiLCJBTiI6Ik1haWwiLCJXVCI6Mn0%3D%7C1000&amp;sdata=%2FCJlewNNBGP89%2FXYmYPDwJN6fXhpxO0Ig2VbLCPzjv4%3D&amp;reserved=0 . **Please note that citations should be in square brackets.**

*We have adjusted the reference style accordingly, not shown in track changes.*

•	**Thank you for providing your [CONSORT/PRISMA/STROBE] checklist. Please replace the page numbers with paragraph numbers per section (e.g. "Methods, paragraph 1"), since the page numbers of the final published paper may be different from the page numbers in the current manuscript.**

*We have revised the STROBE checklist accordingly*

•	**Line 409 -410, the term "trend" is used to refer to a nonsignificant P value. The term trend should be used only when the test for trend has been conducted. Please revise accordingly.**

*We have replaced the term “trend” by “tendency” (line 416)*

**•	Please style reference call outs as follows throughout the text, noting the absence of spaces within the square brackets: "... countries [1,2]."**

*We have adjusted the reference style accordingly.*

•	**In the reference list, please ensure that all citations have full access details, e.g., reference 1.**

*We have corrected reference 1 and double checked all other references*

•	**Please abbreviate journal names consistently, e.g., "PLoS ONE"; "PLoS Med.".**

*We used “Endnote” for referencing and double checked: all PLoS journals are spelled out consistently*
